# MAGMDA: a multi-order adaptive graph-based miRNA-disease association prediction model

**DOI:** 10.1186/s12859-026-06433-z

**Published:** 2026-03-28

**Authors:** Xiujuan Guo, Ning Zhao, Guohua Wang, Chunlong Zhang

**Affiliations:** 1 College of Computer and Control Engineering, Northeast Forestry University, Harbin, 150040 Heilongjiang China; 2 School of Computer Science and Technology, Harbin Institute of Technology, Harbin, 150006 Heilongjiang China

**Keywords:** miRNA-disease association prediction, Adaptive multi-order moment, Dynamic threshold network, Attention mechanism, Hepatocellular carcinoma

## Abstract

**Background:**

MicroRNAs are key biomarkers for human diseases; however, experimentally identifying miRNA-disease associations was costly and inefficient. To improve the robustness and interpretability of computational models, we developed a multi-order adaptive graph-based miRNA-disease association model (MAGMDA).

**Results:**

MAGMDA introduced an adaptive moment-order selection mechanism and a dynamic threshold network, which replaced the fixed moment orders and manually defined thresholds commonly used in traditional high-order statistical modeling with learnable components. By integrating numerically stable high-order moment computation with cross-order attention aggregation, MAGMDA enhanced model robustness and interpretability while maintaining computational efficiency. Five-fold cross-validation on the HMDD v2.0 dataset showed that MAGMDA achieved an AUC of 93.58% and an AUPR of 93.48%. Across multiple evaluation metrics, MAGMDA outperformed representative existing methods, exhibiting particularly consistent advantages on ranking-related metrics such as the area under the receiver operating characteristic curve (AUC) and the area under the precision–recall curve (AUPR), while maintaining smaller performance variations across different cross-validation folds, indicating stable predictive ability under different data partitions. Mechanistic diagnostic analysis further verified the stable utilization of the adaptive modules across different training folds. To demonstrate biological utility, we applied MAGMDA to hepatocellular carcinoma research. Based on the model predictions and analysis of the TCGA-LIHC cohort, we constructed a seven-gene prognostic signature, which was successfully validated in independent datasets and showed strong prognostic stratification ability, highlighting its potential for clinical translation.

**Conclusions:**

MAGMDA effectively improved miRNA-disease association prediction through an adaptive multi-order moment modeling framework and, supported by systematic biological validation, provided a powerful tool for translational research from computational discovery to clinical application. The MAGMDA framework and data resources are publicly available at https://github.com/zhangclbio/MAGMDA.

## Background

MicroRNAs (miRNAs) are a class of endogenous, single-stranded non-coding RNA molecules that mediate post-transcriptional regulation of gene expression by binding to messenger RNAs (mRNAs). They play critical roles in the initiation and progression of multiple complex diseases, including cancer, cardiovascular diseases, metabolic syndrome, and immune-related diseases [1]. An increasing number of studies indicate that dysregulation of miRNA expression is closely associated with disease onset, progression, and prognosis, rendering miRNAs important diagnostic biomarkers and potential therapeutic targets [2]. Therefore, the systematic and accurate identification of miRNA-disease associations (MDAs) facilitates understanding of disease mechanisms at the molecular level and provides insights for early detection, personalized therapy, and prognostic assessment.

MiRNAs have been demonstrated to be closely associated with a wide range of complex diseases. For example, hsa-miR-21 and hsa-miR-155 are persistently overexpressed in multiple cancers, including breast cancer, lung cancer, and hepatocellular carcinoma, and are regarded as typical oncogenic miRNAs [3–5]. The liver-specific hsa-miR-122 is strongly associated with hepatic lipid metabolism disorders and hepatocarcinogenesis [6]. The cardiomyocyte-specific hsa-miR-133 is implicated in cardiovascular events such as myocardial infarction and arrhythmia [7]. hsa-miR-33 and hsa-miR-375 participate in the regulation of lipid metabolism and pancreatic islet function and play important roles in obesity and diabetes [8, 9]. In addition, hsa-miR-146a and hsa-miR-124 are associated with inflammatory responses and neuronal injury in rheumatoid arthritis and neurodegenerative diseases [10, 11]. Collectively, these examples indicate that miRNAs are involved in key pathological processes across multiple biological systems and disease spectra, and that their aberrant expression patterns provide an important foundation for the identification of disease-related molecular biomarkers.

Early studies on MDAs primarily rely on wet-lab techniques such as microarray profiling, real-time quantitative polymerase chain reaction (RT-qPCR), and high-throughput sequencing [12–14]. Although these methods provide high accuracy and reliability at the molecular level, they are often limited by long experimental cycles, high costs, and restricted throughput. As a result, they are not well suited for large-scale screening across an expanding miRNA candidate space and multiple disease contexts. With the rapid development of biomedical public databases and computational science, computational prediction approaches have gradually become an important complement to MDA research. These methods enable rapid global-scale prioritization of massive numbers of candidate miRNA–disease pairs, thereby providing smaller and more informative candidate sets for subsequent experimental validation, which substantially reduces validation costs and shortens research cycles.

Against the backdrop of increasingly abundant data resources, computational approaches based on the functional similarity hypothesis have become one of the important paradigms for predicting MDAs [15]. These methods typically exploit miRNA functional similarity, disease semantic similarity, and known associations to construct similarity networks, and then apply techniques such as learning-to-rank or matrix factorization to predict potential associations. Although these approaches have alleviated the time and cost burdens associated with exclusive reliance on biological experiments, they still suffer from inherent limitations in the deep integration of multi-source information and in modeling complex nonlinear relationships.

To overcome the limitations, researchers further introduce more sophisticated machine learning and deep learning models. The core advantage of these approaches lies in their ability to automatically learn latent feature representations of miRNAs and diseases from data. For example, matrix factorization–based models (e.g., PMFMDA) decompose association matrix to uncover underlying low-dimensional structural information [16], whereas deep neural network–based methods (e.g., DBNMDA) combine unsupervised pretraining with supervised fine-tuning to more fully capture nonlinear features, thereby improving prediction robustness and accuracy [17]. In addition, methods based on semi-supervised learning frameworks (e.g., RLSMDA) are able to simultaneously exploit similarity network structures and sparse known associations for inference, offering certain advantages when labeled information is limited [18]. Although these models achieve notable progress in predictive performance and application efficiency, they still face challenges in the unified modeling of complex topological relationships among biological entities.

In recent years, the emergence of graph neural networks (GNNs) has provided a promising solution to the aforementioned challenges. GNNs are well suited to representing miRNAs, diseases, their similarity relationships, and other biological interactions within a unified graph structure, and enable end-to-end representation learning by jointly integrating topological information and node attributes through neighborhood aggregation mechanisms. Models such as graph convolutional networks (GCNs) and graph attention networks (GATs) have been successfully applied to MDA prediction tasks, demonstrating strong performance in integrating multi-source information and mining high-order topological relationships [19, 20].

However, existing GNN-based MDA prediction methods still leave room for improvement. One key issue is that standard neighborhood aggregation operations (e.g., mean pooling) typically rely on first-order statistics of neighborhood features (i.e., the mean). When different nodes exhibit similar first-order statistics but differ substantially in higher-order properties such as variance and skewness, conventional aggregation schemes may smooth out important discriminative information. This may reduce the representational power of nodes and negatively affect model performance and interpretability. Therefore, how to design aggregation mechanisms that can explicitly characterize and preserve higher-order statistical features of neighborhoods remains an important research question for improving GNN-based applications in bioinformatics.

To characterize the distributional patterns of neighborhood features, recent studies have begun to incorporate higher-order statistics into graph-structured modeling [21]. Related work has shown that constructing multi-order moment features on miRNA–disease heterogeneous graphs, together with mechanisms such as attention and residual connections, can improve association prediction performance to a certain extent. However, such high-order moment–based models typically rely on predefined sets of moment orders and manually specified thresholds. This static strategy is difficult to adapt to the diverse neighborhood distribution characteristics of different nodes in a graph, thereby limiting model expressiveness and generalization ability, and incurring substantial hyperparameter-tuning costs in cross-dataset transfer and practical applications.

It is worth noting that in many existing high-order or multi-scale GNN frameworks, the notion of “high-order” primarily emphasizes topological expansion, such as incorporating multi-hop neighborhood information or combining representations from different receptive-field depths through layer stacking. In contrast, MAGMDA adopts a statistical high-order modeling perspective. Rather than explicitly constructing higher-hop adjacency structures, the proposed model focuses on encoding higher-order statistical properties of local neighborhood feature distributions via moment-based modeling. This distinction allows MAGMDA to capture distributional characteristics beyond mean aggregation, such as variance and higher-order shape information, which are typically overlooked in conventional averaging-based aggregation schemes.

To address these issues, we propose MAGMDA, a miRNA–disease association prediction model based on adaptive multi-order moment modeling. MAGMDA introduces an adaptive moment-order selection mechanism and a dynamic threshold network within the high-order moment encoding module. This design replaces fixed moment orders and manually defined thresholds with a learnable gating strategy. In addition, cross-order attention is used to assign differentiated weights to selected orders, enabling data-driven aggregation. Experimental results demonstrate that MAGMDA achieves stable performance on evaluation metrics such as AUC, while maintaining lower variation across cross-validation folds. Further analysis of the internal mechanisms of the adaptive modules provides empirical evidence of their effectiveness.

Beyond computational performance evaluation, to validate the biological significance and potential clinical translational value of the model predictions, we use hepatocellular carcinoma (HCC) as an application scenario and establish a multi-level validation pipeline spanning from computational prediction to clinical prognosis. This pipeline starts with the set of HCC-related miRNAs predicted by MAGMDA. It then integrates evidence from multiple sources, including external databases, differential expression analysis in the TCGA-LIHC cohort, construction of miRNA–mRNA negative regulatory networks, functional enrichment analysis, and protein–protein interaction analysis. Ultimately, a seven-gene prognostic signature is identified and successfully validated in independent datasets, thereby forming a closed validation loop from computational discovery to molecular mechanisms and clinical outcomes. These results demonstrate the applicability of MAGMDA in identifying disease-related miRNAs and its potential to advance tumor prognosis research.

## Methods

### Data Resources and Preprocessing

This study was based on multiple publicly available data resources. First, experimentally validated miRNA–disease association data were obtained from the Human miRNA-Disease Database (HMDD v2.0; https://www.cuilab.cn/hmdd), including 5430 associations involving 383 diseases and 495 miRNAs, which were used to construct the miRNA-disease prediction model and to evaluate its performance [22]. Second, raw miRNA and mRNA expression data, together with corresponding clinical information for HCC, were downloaded from the TCGA-LIHC project in The Cancer Genome Atlas (TCGA) data portal (https://portal.gdc.cancer.gov) [23]. After sample deduplication and quality control, miRNA and mRNA expression datasets were obtained separately for subsequent biological analysis and prognostic modeling. In addition, for independent dataset validation, the GSE14520 dataset was retrieved from the Gene Expression Omnibus (GEO) database (http://www.ncbi.nlm.nih.gov/geo) [24], and expression data together with clinical follow-up information for the Japanese liver cancer cohort ICGC-LIRI-JP were downloaded from the International Cancer Genome Consortium (ICGC) data portal (https://dcc.icgc.org) [25]. A summary of the sources and sample information of the HCC-related public datasets used in this study is provided in Table 1.

**Table 1:** Overview of public HCC–related datasets used in this study

Dataset/Cohort	Database	Description
TCGA-LIHC	TCGA	Includes 50 adjacent non-tumor liver samples and 369 HCC tumor samples.
GSE14520	GEO	Includes 225 HCC samples and 220 tumor-adjacent liver tissue samples, used for independent dataset validation.
ICGC-LIRI-JP	ICGC	RNA-seq data and clinical information from 212 HCC patients, used for independent dataset validation.

### Disease Semantic Similarity Calculation

In this study, disease semantic similarity was calculated based on the Medical Subject Headings (MeSH) hierarchy. MeSH descriptors corresponding to each disease were obtained from public resources provided by the National Center for Biotechnology Information (NCBI; https://www.ncbi.nlm.nih.gov) [26]. Each disease was then represented as a directed acyclic graph (DAG), in which the disease itself and all its ancestor terms in the MeSH hierarchical structure (i.e., parent nodes and higher-level ancestor nodes) were treated as nodes, and parent–child relationships were modeled as directed edges. On this basis, the semantic similarity between any two diseases was defined, thereby constructing the disease semantic similarity matrix $\:{S}_{\mathrm{sem}}^{D}$.

### miRNA Functional Similarity Construction

To construct the miRNA functional similarity matrix $\:{\mathrm{S}}_{\mathrm{f}\mathrm{u}\mathrm{n}\mathrm{c}}^{M}$, we adopted the miRNA functional similarity (miSIM) method to quantify functional similarities among miRNAs. The underlying assumption of miSIM was that functionally similar miRNAs tended to be associated with similar diseases [15]. The relevant data were obtained from https://www.cuilab.cn/files/images/cuilab/misim.

### GIP Kernel-based Similarity Modeling

Under the assumption that functionally similar miRNAs tended to be associated with phenotypically similar diseases, and vice versa, Gaussian interaction profile (GIP) kernel similarity matrix could be constructed for both diseases and miRNAs [27]. In this study, the GIP similarity matrices were computed once using all experimentally validated MDAs in HMDD v2.0 and were kept fixed across cross-validation folds as prior similarity information.

Taking disease GIP similarity as an example, a disease–miRNA interaction profile matrix was first constructed based on known MDAs. The interaction profile of a disease $\:{d}_{i}\:$was denoted as a vector $\:{\mathrm{y}}_{{d}_{i}}$, whose dimensionality equaled the number of miRNAs. The vector $\:{\mathrm{y}}_{{d}_{i}}$ was a binary vector, in which each element indicated whether the disease was known to be associated with the corresponding miRNA: an associated pair was assigned a value of 1, and a non-associated pair was assigned a value of 0. Subsequently, the GIP kernel similarity between diseases $\:{d}_{i}$ and $\:{d}_{j}$ was defined using the Euclidean distance between their corresponding interaction profile vectors:

$$\:{S}_{\mathrm{G}\mathrm{I}\mathrm{P}}^{D}\left({d}_{i},{d}_{j}\right)=\mathrm{e}\mathrm{x}\mathrm{p}\left(-{\gamma\:}_{D}\parallel\:{\mathrm{y}}_{{d}_{i}}-{\mathrm{y}}_{{d}_{j}}{\parallel\:}_{2}^{2}\right)$$

Here, $\:{\gamma\:}_{D}$ denotes the bandwidth parameter that is automatically determined by the data distribution, and appropriate normalization can mitigate the influence of dataset scale on the range of kernel values. Correspondingly, the miRNA GIP kernel similarity matrix can be constructed in an analogous manner: each miRNA is represented by its interaction profile vector with respect to all diseases, and the same GIP kernel function is used to compute the similarity between any two miRNAs, thereby yielding the miRNA-side GIP similarity matrix.

### Similarity Integration and Node Feature Construction

Because similarity distributions derived solely from the disease semantic similarity matrix $\:{S}_{\mathrm{s}\mathrm{e}\mathrm{m}}^{D}$ and the miRNA functional similarity matrix $\:{S}_{\mathrm{f}\mathrm{u}\mathrm{n}\mathrm{c}}^{M}$ still contained a substantial number of sparse or near-zero entries, we further incorporated GIP kernel–based similarity to complete and smooth these matrices. Specifically, on the disease side, $\:{S}_{\mathrm{s}\mathrm{e}\mathrm{m}}^{D}$was integrated with the disease GIP similarity matrix $\:{S}_{\mathrm{G}\mathrm{I}\mathrm{P}}^{D}$ to obtain a comprehensive disease similarity matrix $\:{I}_{D}$. on the miRNA side, $\:{S}_{\mathrm{f}\mathrm{u}\mathrm{n}\mathrm{c}}^{M}$ was integrated with the miRNA GIP similarity matrix $\:{S}_{\mathrm{G}\mathrm{I}\mathrm{P}}^{M}$ to yield a comprehensive miRNA similarity matrix $\:{I}_{M}$.

In practice, for entries with nonzero semantic or functional similarity, the original information was preferentially retained; for entries with zero similarity or insufficient information, the corresponding GIP similarity values were introduced for completion and smoothing. In this way, the sparsity of the similarity matrix was effectively alleviated while preserving biological prior knowledge. Finally, each row of the integrated similarity matrix $\:{I}_{D}$ and $\:{I}_{M}$ was used as the attribute feature of the corresponding disease node and miRNA node, respectively, and served as part of the node features input to the graph neural network, together with topological embeddings and other information for the MAGMDA model.

### Heterogeneous Graph Construction

In this study, a miRNA–disease heterogeneous graph $\:G=\left(V,E\right)$ was constructed based on known MDAs in HMDD v2.0. The node set consisted of a disease node set$\:\:{\:V}_{D}$ and a miRNA node set $\:{V}_{M}$, with $\:\mid\:{V}_{D}\mid\:=383$ and $\:\mid\:{V}_{M}\mid\:=495$, resulting in a total of 878 nodes. The edge set$\:\:E$ comprised all experimentally validated MDAs. For each known association $\:\left({d}_{i},{m}_{j}\right)$, a directed edge $\:{d}_{i}\to\:{m}_{j}$ from the disease node to the miRNA node was added to the graph, and a reverse edge $\:{m}_{j}\to\:{d}_{i}\:$was also included to enhance information propagation between the two types of nodes. Based on this construction, the adjacency matrix $\:A$ was obtained and further transformed into a normalized adjacency matrix $\:\stackrel{\sim}{A}$ via degree normalization, which was used for message passing and neighborhood statistics computation in subsequent adaptive high-order moment convolution layers. Known MDAs were treated as positive samples in supervised learning, whereas negative samples were randomly drawn from the set of miRNA–disease pairs that are not recorded as experimentally validated associations in HMDD v2.0. In this study, the term “unknown miRNA–disease pairs” refers to pairs that are unconfirmed in the entire curated database, rather than pairs absent from a particular training fold. A balanced sampling strategy was adopted, in which the number of negative samples was matched to the number of positive samples to avoid severe class imbalance. These negative samples were generated once from the global pool of unconfirmed pairs prior to cross-validation and were subsequently combined with the positive samples for five-fold partitioning. These sampled pairs were used as negative instances for model training. During cross-validation, only training-fold associations were used as positive labels for model optimization, while test-fold associations were held out from training.

### Topological Feature Embedding

To explicitly encode the topological information of nodes, we applied the Node2Vec algorithm to learn node embeddings on the miRNA-disease heterogeneous graph [28]. Node2Vec samples sequences of nodes through biased random walks on the graph and trains a Skip-gram model to generate low-dimensional vector representations for each node. In this study, the embedding dimensionality was set to 64 to achieve a balance between representational capacity and computational cost. The resulting Node2Vec embeddings were concatenated with the attribute features provided by $\:{I}_{D}$ and $\:{I}_{M}$ along the feature dimension, and further combined with one-hot encodings of node types. These concatenated features were then linearly projected to obtain the initial node features used in the model.

### MAGMDA Framework and Architecture 

**Fig. 1:**
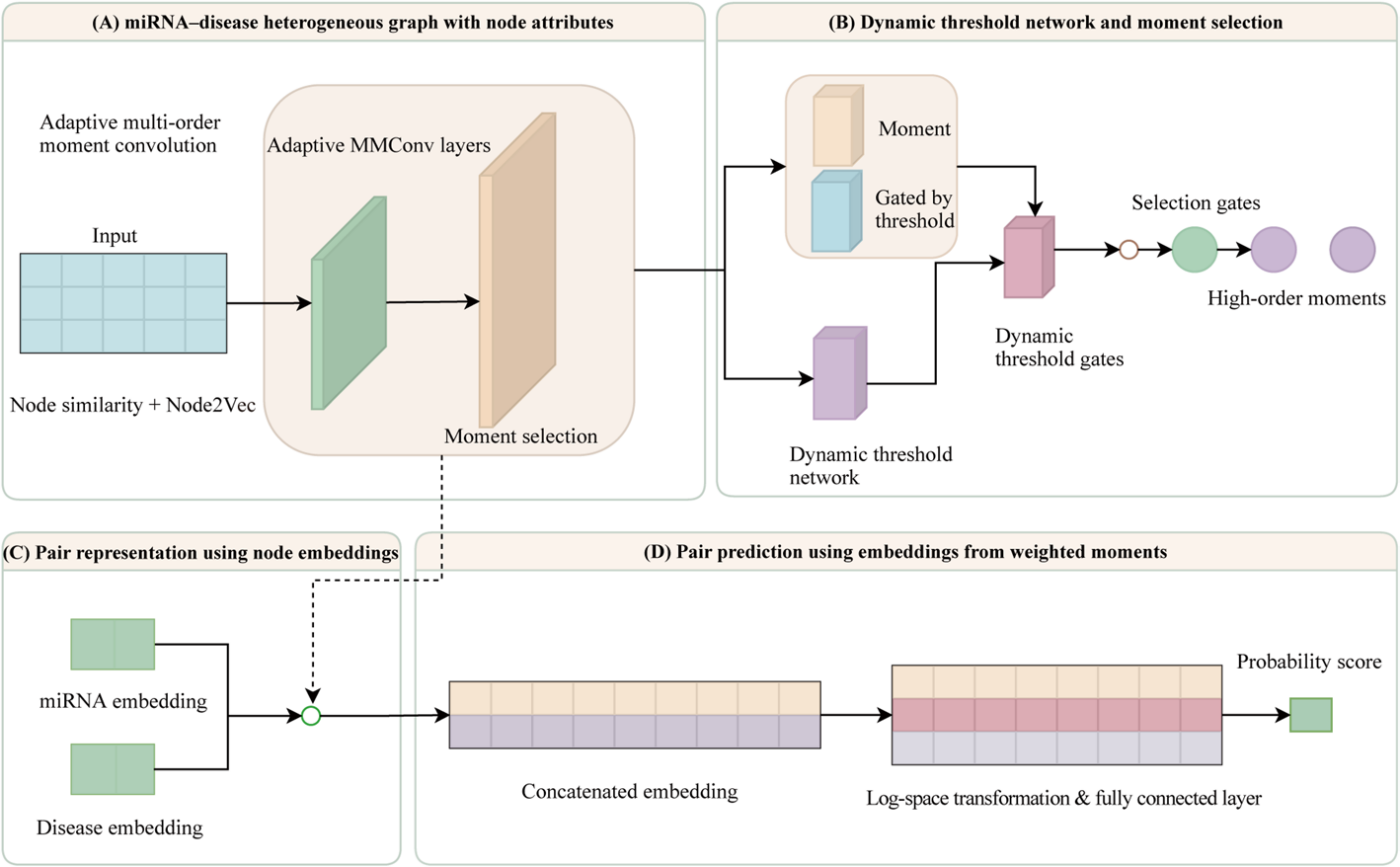
Architecture and prediction workflow of the MAGMDA model. **A** Node feature encoding and adaptive multi-order moment convolution on the miRNA–disease heterogeneous graph. Node inputs consist of integrated similarity features and Node2Vec-based topological embeddings, and updated node representations are obtained through two AdaptiveMMConv layers. **B** The dynamic threshold network and moment-order selection mechanism within AdaptiveMMConv, which generate gating weights for moment features of different orders and adaptively select and weight candidate multi-order moments. **C** Construction of paired representations using disease and miRNA node embeddings, where the embeddings of a given miRNA–disease pair are concatenated along the feature dimension to form a paired vector. **D** The paired vector is fed into a fully connected layer followed by a sigmoid activation function to output the association probability of the miRNA–disease pair, which is used for predicting and ranking potential associations

In this work, “high-order moments” refer to statistical moments of order higher than one, whereas “multi-order modeling” denotes the joint utilization and adaptive selection of multiple moment orders within the proposed MAGMDA framework. The architecture and prediction workflow of the proposed adaptive multi-order moment model, MAGMDA, were illustrated in Fig. 1. The integrated disease similarity matrix $\:{I}_{D}$ and the integrated miRNA similarity matrix $\:{I}_{M}$ constructed in the preceding steps were combined with node type information and Node2Vec-based topological embeddings. These components together formed multi-source node features on the miRNA–disease heterogeneous graph. Subsequently, during the graph representation learning stage, an Adaptive Multi-order Moment Convolutional Layer (AdaptiveMMConv) was introduced. Given a predefined maximum moment order, this layer adaptively selected and weighted neighborhood multi-order moments through moment-order importance parameters, a dynamic threshold network, and cross-order attention. As a result, the updated node representations incorporate high-order statistical information. Finally, in the association prediction stage, disease and miRNA node embeddings were projected into a unified latent space. The embeddings of each disease–miRNA pair were then concatenated and fed into a fully connected layer followed by a sigmoid activation function to obtain the association probability. Model training and performance evaluation were conducted under a unified five-fold cross-validation protocol, and the resulting association probabilities were used to score and rank potential MDAs.

### Adaptive Multi-order Moment Convolutional Layer

In this work, the term moment order consistently refers to the statistical order in moment computation and should not be interpreted as hop-based topological order in graph convolution. During the graph representation learning stage, MAGMDA stacked two AdaptiveMMConv layers to explicitly model multi-order statistical information within node neighborhoods. Let the node feature representation at the $\:l$-th layer be denoted as $\:{\mathrm{H}}^{\left(l\right)}$, where the feature of node $\:i$ is $\:{\mathrm{h}}_{i}^{\left(l\right)}$. Given a predefined maximum moment order $\:{M}_{\mathrm{m}\mathrm{a}\mathrm{x}}$, the model sequentially computes neighborhood high-order moment features from the first order up to the $\:{M}_{\mathrm{m}\mathrm{a}\mathrm{x}}$-th order. In this study, $\:{M}_{\mathrm{m}\mathrm{a}\mathrm{x}}$ was set to 10, serving as an upper-bound candidate pool of statistical orders. This value was chosen by balancing representational expressiveness and numerical stability: while increasing the moment order allows the model to capture richer distributional characteristics of neighborhood features, excessively high-order moments may introduce instability due to power amplification and noise sensitivity in biological datasets. Therefore, $\:{M}_{\mathrm{m}\mathrm{a}\mathrm{x}}\:$=10 was selected as a moderate upper bound. Taking raw moments as an example, the $\:k$-th-order moment of node $\:i$ was computed as follows:

$$\:{\mathrm{m}}_{i,k}^{\left(l\right)}=\sum\:_{j}\:{\stackrel{\sim}{a}}_{ij}{\hspace{0.17em}}({\mathrm{h}}_{j}^{\left(l\right)}{)}^{\odot\:k}\:,\:\:k=1,\dots\:,{M}_{\mathrm{m}\mathrm{a}\mathrm{x}}$$

Here, $\:{\stackrel{\sim}{a}}_{ij}$ denotes the normalized adjacency coefficient, and $\:\odot\:k$ represents the element-wise $\:k$-th power operation.

In practice, neighborhood features were centralized and scaled before computing high-order moments to alleviate numerical instability caused by high-order power operations. For the second-order moment, an approximate variance form was adopted, whereas clipping and logarithmic transformations were applied to enhance numerical stability for higher-order moments.

Based on the high-order moment features, the model further introduced an adaptive moment-order selection mechanism. Specifically, a learnable importance parameter vector $\:\alpha\:$ was assigned to each moment order. Gumbel-Softmax was used to sample $\:\alpha\:$ to obtain soft weights during training, whereas a standard Softmax was applied to yield deterministic weights during inference [29]. Meanwhile, a lightweight threshold network $\:\mathcal{T}\left(\cdot\:\right)$ encoded the overall feature representation of the current layer and output a scalar threshold $\:\tau\:$. Subsequently, a gating coefficient was computed for each moment order $\:k$ as follows:

$$ {\mathrm{g}}_{{\mathrm{k}}} = \sigma \left( {\frac{{{\mathrm{W}}_{{\mathrm{k}}} - \tau }}{\gamma }} \right) $$

Here, $\:\sigma\:\left(\cdot\:\right)$ denotes the sigmoid function, and $\:\gamma\:$ is a temperature hyperparameter. To prevent all moment orders from being simultaneously suppressed, we applied a Top-K retention strategy to keep only the K highest-weighted moment orders (K = 5 in this study). The gating results were then combined with the Top-K mask to generate the final effective weights $\:{\stackrel{\sim}{w}}_{k}$ used to scale the moment features of each order. The weighted multi-order moment features were subsequently fed into cross-order aggregation and node representation updates, thereby enabling adaptive modeling of which moment orders participate in aggregation and the relative contribution of each order.

### Attention-based Aggregation and Node Updating

After obtaining the gated multi-order moment features $\:\{\stackrel{\sim}{{w}_{k}}{\:m}_{i,k}^{\left(l\right)}{\}}_{k=1}^{{M}_{max}}$, MAGMDA adaptively fused statistical information from different moment orders through a cross-order attention mechanism. Specifically, each weighted moment feature was first fed into either a linear transformation or a lightweight feed-forward network and projected into the same latent space as the node representations, yielding a set of candidate moment-order embeddings $\:\left\{{z}_{i,k}^{\left(l\right)}\right\}$. Subsequently, the model computed a scalar score for each node–moment-order pair $\:\left(i,k\right)$, which was normalized across the moment-order dimension using a softmax function to obtain the attention weights $\:{\alpha\:}_{i,k}^{\left(l\right)}$. These weights were used to quantify the relative importance of each moment order for a given node at the current layer [30]. Finally, the representation of node $\:i$ at layer $\:l+1$ was obtained by combining the weighted sum of multi-order moment embeddings with residual information, as follows:

$$ h_{i}^{{({\mathrm{l}} + 1)}} = \sigma \left( {\sum\limits_{{{\mathrm{k}} = 1}}^{{{\mathrm{M}}_{{\max }} }} {\alpha _{{{\mathrm{i,k}}}}^{{({\mathrm{l}})}} {\mathrm{z}}_{{{\mathrm{i,k}}}}^{{({\mathrm{l}})}} + h_{{\mathrm{i}}}^{{({\mathrm{l}})}} } } \right) $$

Here, $\:\sigma\:\left(\cdot\:\right)$ denotes a nonlinear activation function, and $\:{\mathrm{h}}_{i}^{\left(l\right)}$ represents the node embedding from the previous layer. In this way, the attention weights are dynamically allocated across the selected moment orders, enabling the model to distinguish the relative importance of different moments for a given node, rather than treating all orders equally through simple averaging. Meanwhile, the residual connection preserves information from the previous layer, which helps alleviate over-smoothing and gradient vanishing issues [31, 32]. After two layers of adaptive multi-order moment convolution and attention aggregation, disease and miRNA node embeddings are obtained that jointly encode attribute features, topological structure, and high-order statistical information, providing foundational representations for subsequent pair construction and association prediction.

### Association Prediction and Model Training 

After obtaining the final node embeddings, MAGMDA separately extracted the representations of disease nodes and miRNA nodes, denoted as $h_i^D$for disease $\:{d}_{i}$ and $h_j^M$ for miRNA $\:{m}_{j}$, respectively. For any disease–miRNA pair$\:\:({d}_{i},{m}_{j})$, their embeddings were concatenated along the feature dimension to form a paired representation vector ${\mathrm{u}}_{{{\mathrm{ij}}}} = \left[ {h_{i}^{{\mathrm{D}}} ;{\kern 1pt} h_{j}^{{\mathrm{M}}} } \right]$. Subsequently, $\:{u}_{ij}$ was fed into a fully connected layer followed by a sigmoid activation function, producing an association probability between 0 and 1, which was used as the confidence score indicating the existence of a true association between the corresponding miRNA–disease pair:

$$\:{\widehat{y}}_{ij}=\sigma\:\text{{0.17em}}({w}^{\top\:}{u}_{ij}+b)$$

Here, $\:w$ and $\:b$ are learnable parameters, and $\:\sigma\:(\cdot\:)$ denotes the sigmoid function.

The model parameters were optimized by minimizing the binary cross-entropy (BCE) loss function. Given the predicted association probability $\:{\widehat{y}}_{ij}$ for a miRNA–disease pair $\:\:({d}_{i},{m}_{j})$ and its ground-truth label $\:{y}_{ij}\in\:\left\{\mathrm{0,1}\right\}$, the loss function is defined as:

$$\:\mathcal{L}=-\frac{1}{N}{\sum\:}_{\left(i,j\right)}\left[{y}_{ij}\mathrm{log}\left({\widehat{y}}_{ij}\right)+\left(1-{y}_{ij}\right)\mathrm{log}\left(1-{\widehat{y}}_{ij}\right)\right] $$

where $\:\:N$denotes the total number of training samples in the current fold.

Under a unified five-fold cross-validation protocol, the known miRNA–disease associations were randomly partitioned at the association level into five equal subsets. In each fold, four subsets were used as the training set and the remaining subset was used as the test set. During model optimization, only the training-fold associations were treated as positive labels for supervised learning, while test-fold associations were strictly held out and not involved in parameter updating. The pre-sampled negative instances were partitioned into folds together with the positive samples, ensuring that no test-fold labels were used during training. The binary cross-entropy loss was minimized on the training set.

For the test samples, evaluation metrics such as the area under the receiver operating characteristic curve (AUC) and the area under the precision–recall curve (AUPR) were computed based on the predicted probabilities $\:{\widehat{y}}_{ij}$, and these probabilities were further used to score and rank all candidate miRNA–disease pairs.

### Evaluation Metrics

Under the aforementioned five-fold cross-validation setting, the predictive performance of MAGMDA and the comparative models was evaluated. For each test fold, samples in the test set were binarized based on the association probabilities output by the models, and the numbers of true positives (TP), true negatives (TN), false positives (FP), and false negatives (FN) were recorded. Based on these quantities, the following evaluation metrics were calculated:

Accuracy reflects the overall correctness of the predictions and is defined as:

$$\:Accuracy=\frac{TP+TN}{TP+TN+FP+FN}$$

Precision measures the proportion of true positive samples among those predicted as positive by the model and is defined as:

$$\:Precision=\frac{TP}{TP+FP}$$

F1-score is a metric that jointly considers precision and false negatives, and is defined as:

$$\:F1=\frac{2TP}{2TP+FP+FN}$$

In addition, to evaluate the overall discriminative ability and ranking performance of the models under different decision thresholds, we also calculated the AUC and AUPR. Within the five-fold cross-validation framework, these metrics were computed separately for the test set in each fold, and the mean values and standard deviations across the five folds were reported as the final results, thereby characterizing the overall performance and stability of the models.

### Implementation Details

The proposed MAGMDA model was implemented in Python (version 3.6.5) using PyTorch (version 1.7.1 + cu110). All experiments were conducted on a workstation equipped with an NVIDIA GeForce RTX 3060 GPU. To ensure reproducibility, a fixed random seed (1234) was used throughout all experiments.

The main hyperparameters were configured as follows: the optimizer was Adam with a learning rate of 5 × 10⁻⁵ and weight decay of 1 × 10⁻³. The hidden dimension size was set to 64, and the model consisted of two GNN layers. The maximum candidate moment order was set to 10, with a Top-K retention size of 5. Dropout rates were set to 0.3 for the main dropout and 0.2 for node-level and feature-level dropout.

All downstream statistical analyses were performed using R (version 4.5.2), including DESeq2 (version 1.46.0) for differential expression analysis, survival (version 3.8.3) and timeROC (version 0.4) for prognostic evaluation, and clusterProfiler (version 4.14.6) for functional enrichment analysis.

### HCC-related miRNA Prediction and Validation

After model training, HCC was used as a case study to identify candidate HCC-related miRNAs from the predictions of MAGMDA and to validate them using external databases. Specifically, in the constructed miRNA–disease heterogeneous graph, the disease node corresponding to HCC was identified and denoted as $\:{d}_{\mathrm{H}\mathrm{C}\mathrm{C}}$. Using the trained MAGMDA model, this disease node was paired with all miRNA nodes $\:\left\{{m}_{j}\right\}{\:}_{j=1}^{495}$, and the association probability for each pair $\:({d}_{\mathrm{H}\mathrm{C}\mathrm{C}},{m}_{j})$ was computed, denoted as $\:{\widehat{y}}_{\mathrm{H}\mathrm{C}\mathrm{C},j}$, which served as the model-predicted confidence score for the association between the miRNA and HCC. All $\:{\widehat{y}}_{\mathrm{H}\mathrm{C}\mathrm{C},j}$ values were then ranked in descending order to obtain a prioritized list of HCC-related miRNAs. In this study, the top 50 miRNAs were selected as a high-confidence candidate set of HCC-associated miRNAs.

To assess the extent of external evidence supporting the candidate miRNAs, we further queried the miRNA differential expression database dbDEMC (http://www.picb.ac.cn/dbDEMC) to retrieve information on the top 50 candidate miRNAs (ranked by prediction score) [33]. This step was performed solely as an independent validation of the model predictions and was not involved in the training or parameter estimation of the MAGMDA model.

### Differential Expression Analysis

Based on the miRNA and mRNA expression data from the TCGA-LIHC project, samples were divided into a tumor group and an adjacent normal tissue group according to tissue origin. After data quality control and expression normalization, differential expression analysis was performed separately for miRNAs and mRNAs. Specifically, for each expression feature (miRNA or mRNA), the R package DESeq2 [34] was used to assess expression differences between tumor and normal groups, yielding the corresponding log2 fold change (log2FC) and *P* value. In this study, features with *P* value ≤ 0.05 and |log2FC| ≥ 1 were considered differentially expressed. Based on these criteria, sets of differentially expressed miRNAs (DE-miRNAs) and differentially expressed mRNAs (DE-mRNAs) were identified and used for subsequent analysis.

### Key miRNA Identification and Regulatory Analysis

The top 50 HCC-related miRNAs predicted by the model were intersected with the set of DE-miRNAs, yielding a set of key miRNAs that were both predicted by the model and significantly differentially expressed in the TCGA-LIHC cohort. This set was used for subsequent target gene prediction and network construction. It should be emphasized that the following external databases were not involved in model training or parameter optimization. Instead, they were queried only after model prediction as independent reference resources to provide supporting evidence for the predicted miRNA candidates. Subsequently, each key miRNA was queried against miRDB [35] (https://mirdb.org), miRTarBase [36] (https://mirtarbase.cuhk.edu.cn), and TargetScan [37] (https://www.targetscan.org) to retrieve predicted and experimentally validated target gene information. The results obtained from these databases were merged and deduplicated to generate a candidate target gene set for each miRNA. To ensure that subsequent analysis focused on genes exhibiting expression changes in HCC, the candidate target gene sets were intersected with the set of DE-mRNAs, resulting in a candidate set of HCC-related target genes.

To characterize the potential negative regulatory relationships between miRNAs and candidate target genes, Spearman correlation coefficients were calculated between each key miRNA and its corresponding candidate target genes. The *P* value was adjusted using the Benjamini–Hochberg method [38] to obtain the corresponding false discovery rates (FDRs). Based on the biological assumption that upregulated miRNAs tended to downregulate their target genes, miRNA–mRNA pairs with a correlation coefficient of $\:\rho\:\le\:-0.20$ and an FDR ≤ 0.05 were selected as thresholds. Only significantly negatively correlated miRNA–mRNA pairs were retained as high-confidence candidate regulatory relationships for subsequent regulatory network construction and functional analysis.

### Construction of the miRNA–mRNA Regulatory Network

The significantly negatively correlated miRNA–mRNA pairs identified above were used as the edge set, with key miRNAs and their candidate target genes serving as two distinct types of nodes, to construct a miRNA–mRNA regulatory network. In this network, each edge represented a miRNA–mRNA pair that exhibited a significant negative correlation in the TCGA-LIHC cohort and simultaneously satisfied the dual criteria of model prediction support and significant differential expression. This network was used to globally characterize the potential regulatory patterns of key miRNAs on HCC-related gene and to provide gene sets for subsequent functional analysis.

### Functional Enrichment Analysis

The functional enrichment analysis were performed using the Enrichr platform [39] (https://maayanlab.cloud/Enrichr), including Gene Ontology (GO) [40] and Kyoto Encyclopedia of Genes and Genomes (KEGG) [41] pathway analysis. A threshold of *P* value ≤ 0.05 was used to define statistically significant enrichment.

### Protein–protein Interaction Network Construction

The screened candidate HCC-related gene set was used as input and uploaded to the online protein–protein interaction database STRING [42] (https://string-db.org), with the organism restricted to *Homo sapiens*. In the parameter settings, the minimum interaction confidence score was set to 0.7, and evidence derived from text mining was excluded to ensure the reliability of network interactions. From the interaction results returned by STRING, edges that did not meet the confidence threshold and completely isolated nodes were removed, yielding a high-confidence PPI network of candidate HCC-related genes.

To identify hub genes occupying key positions in the network topology, we comprehensively evaluated gene importance based on multiple centrality measures of nodes in the PPI network. Specifically, degree centrality, betweenness centrality, closeness centrality, eigenvector centrality, PageRank score, and k-core value were calculated for each node. These metrics characterize the topological roles of genes from multiple perspectives, including interaction connectivity, information flow, and local–global network structure. Subsequently, each centrality metric was rank-transformed, and the average rank across all metrics was used as an integrated topological score to rank all genes. Finally, hub genes in the PPI network were selected in descending order of this integrated score for subsequent prognostic model construction and biological interpretation.

### Prognostic Model Construction

Based on the identified hub genes, a multi-gene prognostic signature was constructed in the TCGA-LIHC cohort. First, univariate Cox proportional hazards regression analysis was performed for each hub gene [43], and genes with *P* value ≤ 0.05 were retained for subsequent modeling. The least absolute shrinkage and selection operator (LASSO) Cox regression method [44] was then applied to construct the most appropriate gene signature. The optimal penalty parameter was selected using the “lambda.min” criterion implemented in the R package glmnet, resulting in a gene signature set $\:G$ with non-zero regression coefficients. According to the regression coefficients $\:{\beta\:}_{g}$ obtained from the LASSO–Cox model, a risk score was calculated for each patient as follows:

$$\:\mathrm{Risk\:Score}=\sum\:_{g\in\:G}\:{\beta\:}_{g}\times\:{X}_{g}$$

Here, $\:{X}_{g}$ denotes the standardized expression level of gene $\:g$. Patients were divided into high- and low-risk groups according to the median risk score. Kaplan–Meier survival analysis was performed using the survival and survminer packages [45] to examine the association between survival time and risk score. ROC analysis was conducted using the R package timeROC [46] to evaluate the predictive accuracy of the prognostic signature at different follow-up time points. In addition, a nomogram was constructed by integrating the risk score with clinical variables such as age, sex, and tumor stage, to predict the overall survival probability of individual patients at different follow-up times.

### Molecular Subtyping and Clinical Characterization

Based on the expression matrix of the model genes, consensus clustering analysis was performed using the R package ConsensusClusterPlus [47] to identify potential molecular subtypes of HCC. To evaluate the clinical relevance of the identified subtypes, Kaplan–Meier survival curves were generated with subtype grouping as the independent variable, and overall survival differences among subtypes were compared using the log-rank test [48]. Meanwhile, age, sex, and tumor stage were jointly included as covariates in a multivariate Cox proportional hazards regression model to assess the independent prognostic value of the molecular subtypes.

After confirming significant survival differences between subtypes, gene set enrichment analysis (GSEA) [49] was conducted to further elucidate the underlying biological mechanisms. Specifically, genes were pre-ranked based on moderated t statistics derived from limma differential analysis of the genome-wide expression matrix, and pathway-level functional differences between the two subtypes were assessed. Pathways with an FDR ≤ 0.05 were regarded as significantly enriched.

### Immune Infiltration and Immune Checkpoint Analysis

The CIBERSORT algorithm [50] was applied to deconvolute the RNA-seq data of the TCGA-LIHC cohort based on the LM22 gene signature matrix, in order to estimate the relative abundances of 22 immune cell subtypes. Differences in immune cell infiltration levels among different molecular subtypes were compared using the Wilcoxon rank-sum test [51], and an FDR ≤ 0.05 was considered statistically significant. To characterize the overall immunosuppressive status, four immune checkpoint genes—*PDCD1*, *CTLA4*, *LAG3*, and *TIGIT*—were selected and compared across different subtypes.

### Independent Dataset Validation

This study employed the GSE14520 and ICGC-LIRI-JP datasets to further validate the prognosis of the model-derived genes. Using the genes identified from prognostic model construction and molecular subtype analysis as candidate features, together with gene expression matrix and follow-up information from each cohort, multivariate Cox regression models were reconstructed separately in each dataset. Patients were then stratified into high- and low-risk groups according to the calculated risk scores. Kaplan–Meier survival curves and time-dependent ROC curves were generated to evaluate the survival stratification ability and predictive performance of the model in independent populations.

## Results

### Overall Predictive Performance of MAGMDA

Based on the proposed adaptive multi-order moment modeling framework, MAGMDA jointly characterized node attributes, graph topology, and multi-order neighborhood statistical features on the miRNA–disease heterogeneous graph, enabling end-to-end prediction of potential miRNA–disease associations. The predictive performance of MAGMDA was systematically evaluated on the HMDD v2.0 dataset using five-fold cross-validation. The results showed that MAGMDA achieved strong and robust performance in the MDA prediction task: the average AUC and AUPR were 0.9358 ± 0.0044 and 0.9348 ± 0.0059, respectively, while the accuracy, precision, and F1-score were 0.8602 ± 0.0086, 0.8594 ± 0.0140, and 0.8604 ± 0.0079, respectively. The low standard deviations across all metrics indicated that the model was stable under different data splits and exhibited good generalization ability.

**Fig. 2:**
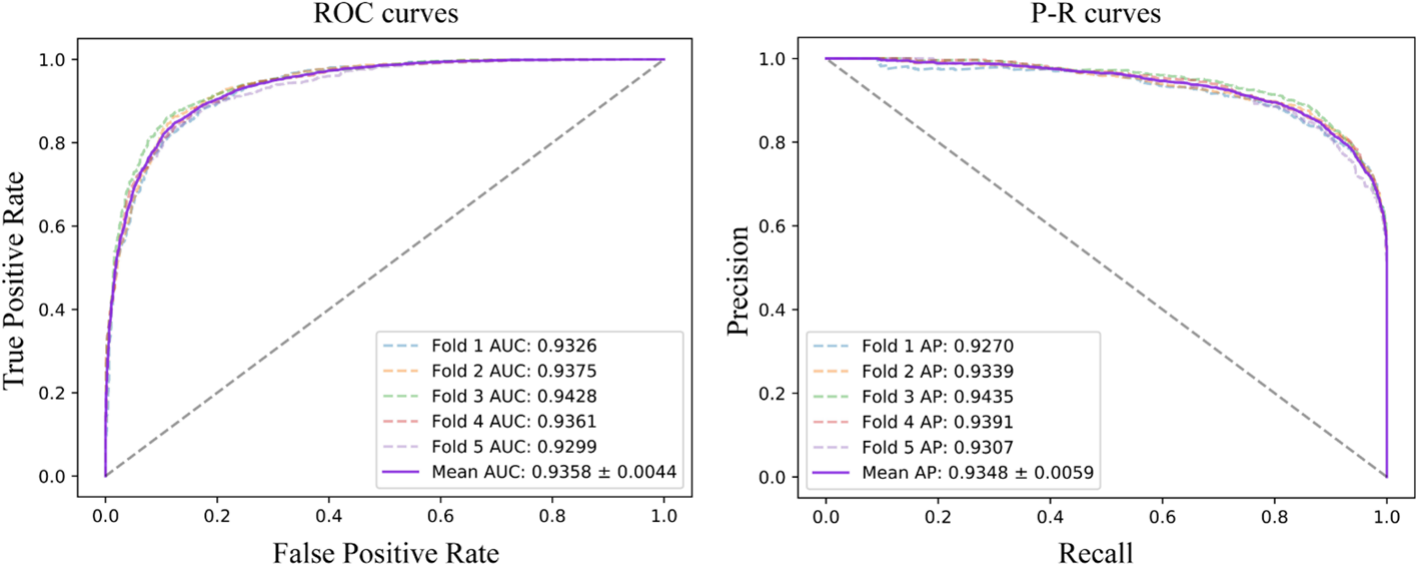
Five-fold cross-validation of MAGMDA. **A** ROC curves performed by MAGMDA. **B** P-R curves performed by MAGMDA

From the perspective of individual folds, the AUC values across the five folds were 0.9326, 0.9375, 0.9428, 0.9361, and 0.9299, respectively, indicating a relatively small overall performance fluctuation. Among them, the third fold achieved the best performance (AUC = 0.9428); therefore, the model trained on this fold was selected as the pretrained model for subsequent HCC-related downstream analysis. As shown in Fig. 2, the ROC and PR curves across different folds exhibited highly similar overall shapes, consistently demonstrating that MAGMDA was able to maintain a high true positive rate and achieve a favorable precision–recall balance across different decision thresholds.

Based on the aforementioned performance metrics, it could be observed that the introduction of adaptive multi-order moment modeling and attention-based aggregation enabled the model to achieve high and stable predictive performance on the HMDD v2.0 dataset, thereby laying a solid foundation for subsequent comparative analysis with other models.

### Performance Comparison with Baseline Models

On the HMDD v2.0 dataset, under the same data partitioning strategy and evaluation metrics, we systematically compared MAGMDA with several representative miRNA–disease association prediction methods. The average performance of five models—MRSLA [52], AGAEMD [53], ABMDA [54], HHOMR [55], and MAGMDA—under five-fold cross-validation on HMDD v2.0 was summarized in Table 2. MAGMDA achieved higher AUC and AUPR values than existing leading methods, demonstrating robust discriminative power in ranking potential miRNA–disease associations. Meanwhile, with respect to classification metrics such as Accuracy, Precision, and F1-score, MAGMDA performed comparably to the existing methods without notable performance decline.

Taken together, while maintaining predictive performance comparable to existing methods, MAGMDA demonstrated consistent advantages in ranking performance and stability, providing a reliable modeling foundation for subsequent analysis of the adaptive multi-order moment modeling mechanism and biological application validation.

**Table 2:** Performance (%) of five methods for MDA prediction on the HMDD v2.0 dataset

Model	AUC.	Acc.	Pre.	F1.	AUPR.
MRSLA	89.45	83.40	83.59	83.34	89.47
AGAEMD	92.70	85.02	84.81	85.07	92.86
ABMDA	91.52	84.39	83.98	84.02	90.69
HHOMR	93.28	**86.25**	85.54	85.67	92.97
**MAGMDA**	**93.58**	86.02	**85.94**	**86.04**	**93.48**

The best-performing results in each category are highlighted in bold

### Mechanistic Analysis of the Adaptive Multi-order Moment Module

**Fig. 3:**
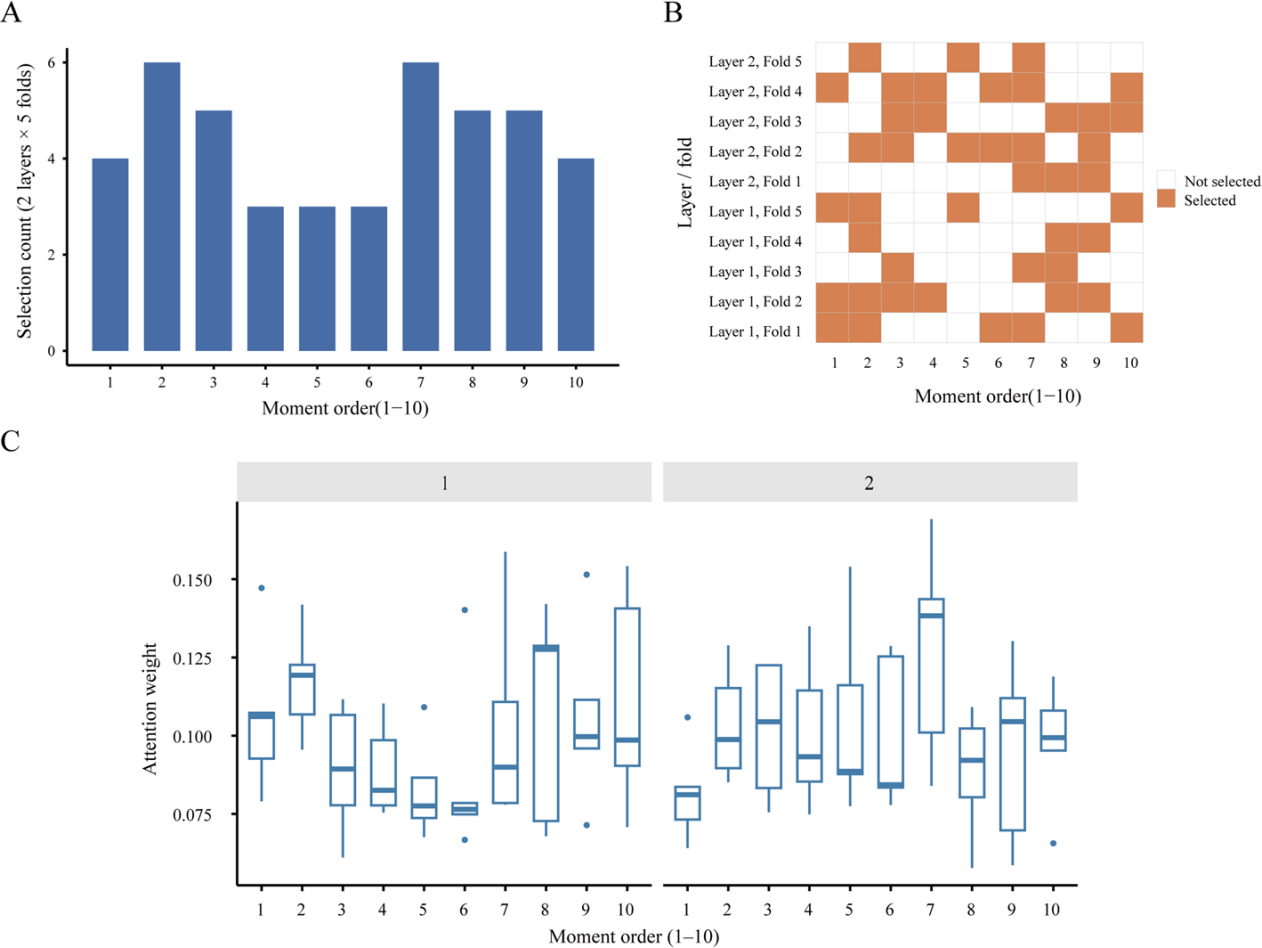
Adaptive moment-order selection and attention allocation in MAGMDA. **A** Distribution of selection frequencies of different-order moments in the adaptive high-order moment module. The x-axis represents the candidate moment orders, and the y-axis represents the cumulative number of times each order was selected during five-fold cross-validation of the two-layer convolutional network. The bar height reflects the usage frequency of each moment order in the model. **B** Heatmap visualization of moment-order selection patterns across different folds and network layers. The x-axis represents the candidate moment orders, and the y-axis represents combinations of network layers and cross-validation folds. Orange squares indicate that the corresponding moment order was selected for aggregation in that layer and fold, while blank cells indicate that it was not selected. **C** Distribution of attention weights for different moment orders in the two-layer MAGMDA model. The x-axis represents the moment orders, and the y-axis represents the corresponding attention weights. The left and right subplots show the statistical distributions of moment-order weights in the adaptive multi-order moment convolutions of Layer 1 and Layer 2, respectively

To validate the effectiveness of the adaptive multi-order moment module, we performed a statistical analysis of the moment-order selection patterns in the two-layer AdaptiveMMConv. Figure 3A illustrated the frequency distribution of selected moment orders across all cross-validation folds. With the maximum moment order set to $\:{M}_{\mathrm{m}\mathrm{a}\mathrm{x}}=10$, which serves as an upper-bound candidate pool rather than a fixed number of utilized orders, the model selected only approximately 4.4 moment orders per layer on average. This indicated that the model neither indiscriminately used all candidate moment orders nor discarded high-order moments; instead, it performed adaptive multi-order selection by dynamically screening the moment orders most relevant to the task. Specifically, the second- and seventh-order moments were selected most frequently (six times each), followed by the third-, eighth-, and ninth-order moments (five times each). The fourth-, fifth-, and sixth-order moments appeared slightly less frequently, whereas the first- and tenth-order moments were each selected four times. This distribution pattern suggested that the model adopted a multi-order modeling strategy, jointly leveraging low-order moments together with a selected subset of mid- to high-order moments to characterize neighborhood feature distributions.

Furthermore, Fig. 3B presented a heatmap illustrating the moment-order selection patterns across different cross-validation folds and network layers. It can be observed that the adaptive module exhibited similar selection behaviors under different data partitions: both convolutional layers consistently focused on a limited number of specific moment orders, rather than showing large random fluctuations across folds. This consistency indicated that the model’s moment-order preferences were not driven by training randomness, but instead reflected relatively stable neighborhood distribution characteristics inherent in the data.

We also analyzed cross-order attention weights. The box plots in Fig. 3C show the distribution of attention weights across moment orders. Frequently selected orders through Top-K mechanism generally received higher attention weights, while less selected orders showed lower weights overall. This aligned with moment-order selection statistics, indicating that the adaptive gating and attention mechanisms work coordinately: unimportant orders were first filtered out, then fine-grained weights were assigned among retained key orders. The adaptive multi-order moment module not only delivered robust performance gains but also confirmed the model’s effective use of multi-order statistical information.

### Prediction and Validation of HCC-related miRNA

Hepatocellular carcinoma (HCC) is one of the malignant tumors with high incidence and mortality worldwide. Its initiation and progression are characterized by pronounced molecular heterogeneity, and miRNAs are widely recognized to play critical roles in the regulation of hepatocarcinogenesis, tumor progression, and prognosis [56]. Therefore, HCC has become a representative disease for evaluating the biological validity and clinical applicability of miRNA–disease association prediction models. To validate the prediction results for HCC, we queried the dbDEMC database for the top 50 miRNAs prioritized by MAGMDA. The results showed that all 50 candidate miRNAs were demonstrated to be associated with HCC in dbDEMC (Table 3). These results indicated that the MAGMDA model was able to effectively identify miRNAs closely associated with HCC, thereby providing a high-confidence set of candidate molecules and a reliable starting point for subsequent analysis.

**Table 3:** Top 50 miRNAs associated with hepatocellular carcinoma predicted by the MAGMDA model

Rank	miRNA	Evidence	Rank	miRNA	Evidence
1	hsa-mir-143	dbDEMC	26	hsa-mir-302d	dbDEMC
2	hsa-mir-133a	dbDEMC	27	hsa-mir-424	dbDEMC
3	hsa-mir-9	dbDEMC	28	hsa-mir-452	dbDEMC
4	hsa-mir-34b	dbDEMC	29	hsa-mir-186	dbDEMC
5	hsa-mir-27b	dbDEMC	30	hsa-mir-625	dbDEMC
6	hsa-mir-206	dbDEMC	31	hsa-mir-215	dbDEMC
7	hsa-mir-429	dbDEMC	32	hsa-mir-32	dbDEMC
8	hsa-mir-193b	dbDEMC	33	hsa-mir-185	dbDEMC
9	hsa-mir-132	dbDEMC	34	hsa-mir-153	dbDEMC
10	hsa-mir-23b	dbDEMC	35	hsa-mir-28	dbDEMC
11	hsa-mir-137	dbDEMC	36	hsa-mir-449a	dbDEMC
12	hsa-mir-342	dbDEMC	37	hsa-mir-339	dbDEMC
13	hsa-mir-196b	dbDEMC	38	hsa-mir-95	dbDEMC
14	hsa-mir-204	dbDEMC	39	hsa-mir-494	dbDEMC
15	hsa-mir-135b	dbDEMC	40	hsa-mir-211	dbDEMC
16	hsa-mir-302a	dbDEMC	41	hsa-mir-363	dbDEMC
17	hsa-mir-26b	dbDEMC	42	hsa-mir-663a	dbDEMC
18	hsa-mir-708	dbDEMC	43	hsa-mir-638	dbDEMC
19	hsa-mir-128	dbDEMC	44	hsa-mir-184	dbDEMC
20	hsa-mir-149	dbDEMC	45	hsa-mir-376c	dbDEMC
21	hsa-mir-194	dbDEMC	46	hsa-mir-520 h	dbDEMC
22	hsa-mir-30e	dbDEMC	47	hsa-mir-103b	dbDEMC
23	hsa-mir-328	dbDEMC	48	hsa-mir-520c	dbDEMC
24	hsa-mir-574	dbDEMC	49	hsa-mir-151b	dbDEMC
25	hsa-mir-367	dbDEMC	50	hsa-mir-371a	dbDEMC

### Identification of Key miRNAs and Construction of the miRNA–mRNA Regulatory Network

**Fig. 4:**
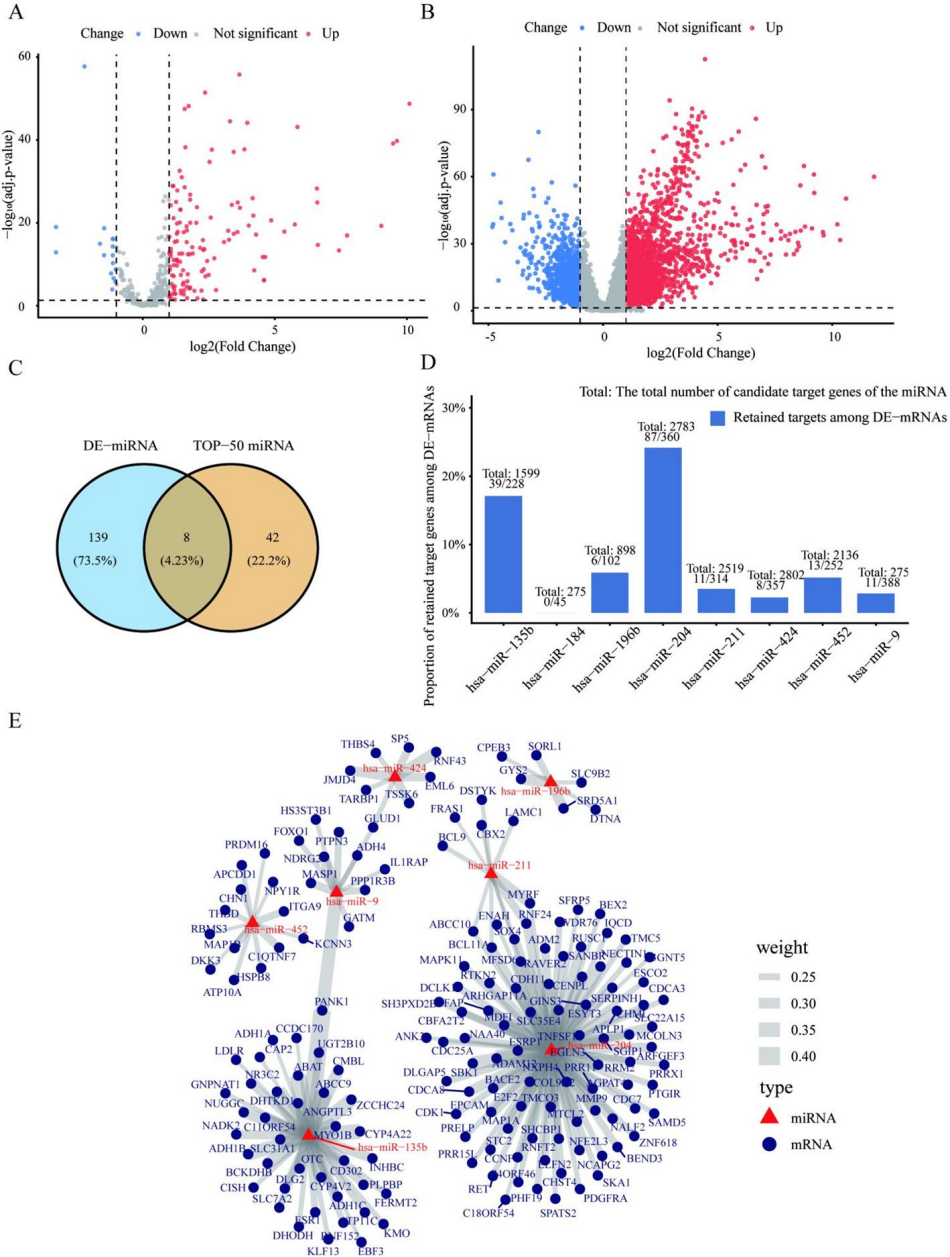
Identification of key miRNAs and construction of the miRNA–mRNA regulatory network in HCC. **A** Volcano plot of DE-miRNAs in the TCGA-LIHC cohort. **B** Volcano plot of DE-mRNAs in the TCGA-LIHC cohort. **C** Intersection between the top 50 miRNAs and 8 DE-miRNAs. **D** Screening results of candidate target genes for key miRNAs. The bar chart shows the total number of candidate target genes for each key miRNA, as well as the number and proportion of those targets that are significantly DE-mRNAs in the TCGA-LIHC cohort. **E** Schematic diagram of the miRNA–mRNA negative regulatory network. Triangular nodes represent miRNAs, and circular nodes represent mRNAs. Each edge in the network indicates a significant negative correlation between the corresponding miRNA and mRNA. Edge thickness reflects the strength of the regulatory relationship, with thicker edges indicating stronger negative correlations

In the TCGA-LIHC cohort, DE-miRNAs and DE-mRNAs were identified using thresholds of FDR ≤ 0.05 and |log₂FC| ≥ 1 (Fig. 4A and B**)**. By intersecting the top 50 HCC-related miRNAs predicted by MAGMDA with the set of DE-miRNAs, eight key miRNAs were obtained (Fig. 4C). These miRNAs included hsa-miR-9, hsa-miR-196b, hsa-miR-204, hsa-miR-135b, hsa-miR-184, hsa-miR-452, hsa-miR-211, and hsa-miR-424.

To further validate the potential biological roles of these eight miRNAs in HCC we retained target genes that were significantly differentially expressed in the TCGA-LIHC cohort. 45–388 candidate target genes were retained for each miRNA (Fig. 4D). We next calculated the correlation coefficients between miRNAs and mRNAs. Following filtering with thresholds of ρ ≤ −0.20 and FDR ≤ 0.05, seven miRNAs retained significantly negatively correlated target genes, whereas hsa-miR-184 did not meet these criteria. Ultimately, 175 significant negative regulatory relationships between 7 miRNAs and 167 target genes were identified, based on which the miRNA–mRNA regulatory network was constructed (Fig. 4E**)**.

To evaluate the robustness of the selected correlation threshold, additional sensitivity analyses were conducted using alternative cutoffs (ρ ≤ −0.15 and ρ ≤ −0.25) while keeping the FDR threshold fixed at 0.05. As expected, relaxing the threshold to ρ ≤ −0.15 increased the number of retained pairs to 308 (275 unique target genes), whereas adopting a more stringent cutoff of ρ ≤ −0.25 reduced the network to 89 pairs (87 target genes). Despite these variations in network density, the regulatory pattern remained stable. Major miRNAs such as hsa-miR-204 and hsa-miR-135b were consistently retained across all thresholds. Notably, hsa-miR-204 remained the dominant regulator under all settings, with 132, 87, and 48 retained targets, respectively. Furthermore, seven of the eight candidate miRNAs were preserved even under the most stringent cutoff, indicating that the core regulatory module is robust to reasonable variations in correlation stringency.

Notably, hsa-miR-204 had the largest number of high-confidence target genes, totaling 87. Previous studies have suggested that this miRNA was involved in key molecular regulatory processes associated with HCC [57]. The identification of hsa-miR-204 by MAGMDA further supported its potential importance within the molecular regulatory network of HCC.

### Functional Enrichment Aanalysis of Target Genes

**Fig. 5:**
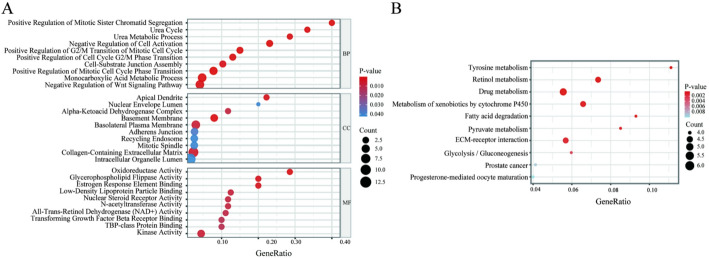
Functional and pathway enrichment analysis of candidate HCC-related target genes. **A** GO enrichment analysis (BP, CC, MF) of 167 genes. The x-axis represents the GeneRatio; bubble size indicates the number of enriched genes, and color intensity reflects the *P*-value. The top 10 terms are shown. **B** KEGG pathway enrichment analysis of 167 genes. The x-axis represents the GeneRatio; bubble size indicates the number of enriched genes, and color intensity reflects the *P*-value. The top 10 pathways are shown

Using the 167 HCC-related target genes in the miRNA–mRNA regulatory network as the background set, GO enrichment analysis was performed. As shown in Fig. 5A, a total of 124 significantly GO terms were identified in the biological process (BP) category, while 10 and 36 significant terms were obtained in the cellular component (CC) and molecular function (MF) categories, respectively (*P* value ≤ 0.05). The top 10 terms in each category ranked by significance were displayed in the figure. The GO enrichment results indicated that, at the BP level, the genes were mainly enriched in cell cycle– and metabolism-related processes, such as positive regulation of mitotic sister chromatid separation, positive regulation of the G2/M phase transition, monocarboxylic acid metabolic processes, and the urea cycle. At the CC level, enriched terms included basement membrane, adherens junction, collagen-containing extracellular matrix, recycling endosome, and mitotic spindle. In the MF category, the enriched functions were primarily associated with oxidoreductase activity, kinase activity, transforming growth factor-β (TGF-β) receptor binding, steroid receptor activity, and lipoprotein binding.

The results of KEGG pathway enrichment analysis were shown in Fig. 5B. Using a significance threshold of *P* value ≤ 0.05, a total of 20 KEGG pathways were identified, among which the top 10 pathways ranked by significance were displayed in the figure. The candidate genes were mainly enriched in multiple metabolism-related pathways, including tyrosine metabolism, retinol metabolism, drug metabolism, and cytochrome P450–mediated metabolism of xenobiotics. In addition, ECM–receptor interaction and several cancer-related pathways also showed significant enrichment, suggesting that these genes might jointly participate in metabolic reprogramming and tumor microenvironment remodeling in HCC.

### Analysis of PPI Network 

**Fig. 6:**
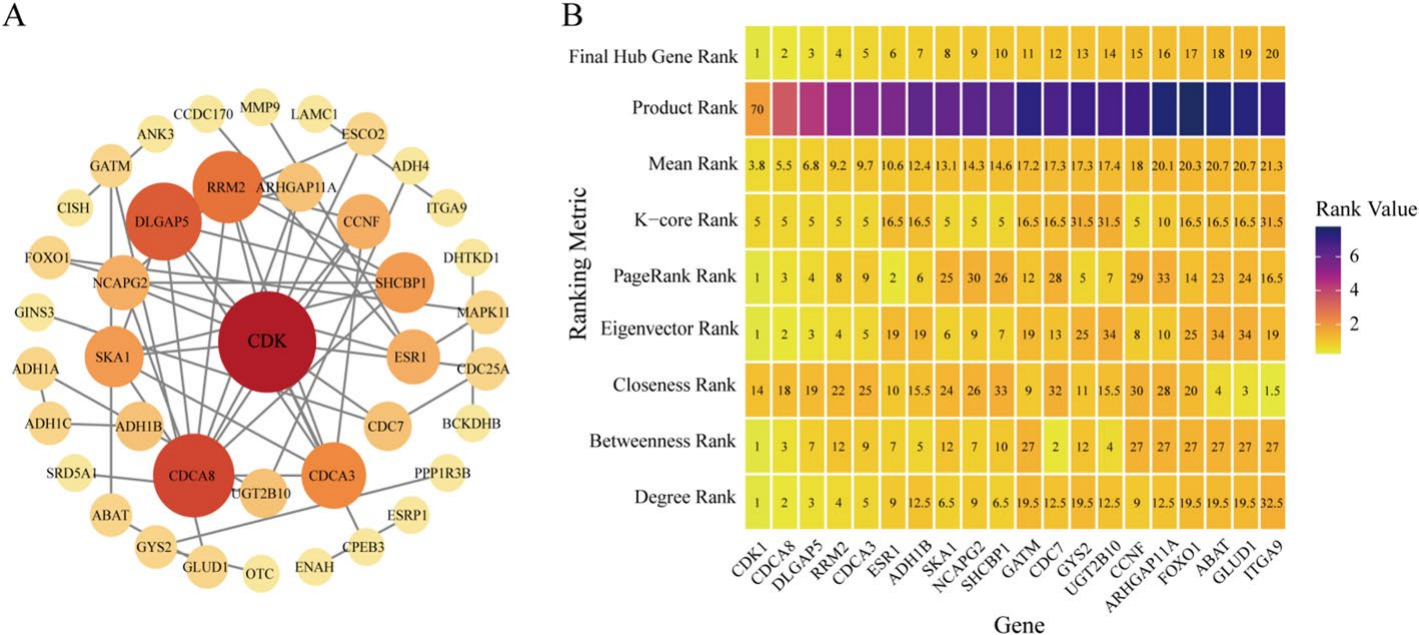
Protein–protein interaction network and identification of hub genes associated with HCC. **A **Based on the candidate gene set, a PPI network was constructed, containing 40 nodes and 56 edges. **B** A heatmap was generated based on six network topological centrality measures and their integrated ranking results. The heatmap displays the individual rankings derived from six centrality metrics—degree, betweenness, closeness, eigenvector, PageRank, and k-core—in the HCC PPI network, as well as the corresponding mean rank, product rank, and final hub gene rank. This provides a systematic characterization of the hierarchical topological importance of candidate hub genes

miRNAs primarily exert their regulatory functions by interacting with their target mRNAs during cancer initiation and progression. Therefore, this study focused on further analyzing mRNAs that potentially interact with key miRNAs. Based on the mRNA set identified above as having potential interactions with miRNAs, we constructed a corresponding PPI network using the STRING database. This PPI network comprised 40 nodes and 56 interaction edges (Fig. 6A**)**.

Based on the PPI network, we calculated six network centrality measures, including degree, betweenness, closeness, eigenvector, PageRank, and k-core [58]. To integrate these multidimensional features, each metric was ranked independently. When multiple genes shared identical values for a given metric, ties were handled using the average ranking method. On this basis, the mean rank and the product rank across the six centrality measures were calculated for each gene. The mean rank was used to comprehensively evaluate the overall topological importance of genes across multiple network features, whereas the product rank served as an auxiliary metric to reflect the consistency of gene rankings among different centrality measures. Finally, genes were ordered in ascending order of the mean rank to derive the final hub gene ranking, based on which the top 20 hub genes were identified. These genes included *CDK1*, *CDCA8*, *DLGAP5*, *RRM2*, *CDCA3*, *ESR1*, *ADH1B*, *SKA1*, *NCAPG2*, *SHCBP1*, *GATM*, *CDC7*, *GYS2*, *UGT2B10*, *CCNF*, *ARHGAP11A*, *FOXO1*, *ABAT*, *GLUD1*, and *ITGA9*. A comprehensive ranking heatmap constructed based on the six network topological features systematically illustrated the hierarchical structure and relative importance of these hub genes within the PPI network (Fig. 6B**)**.

### Construction and Evaluation of Prognostic Signature

**Fig. 7:**
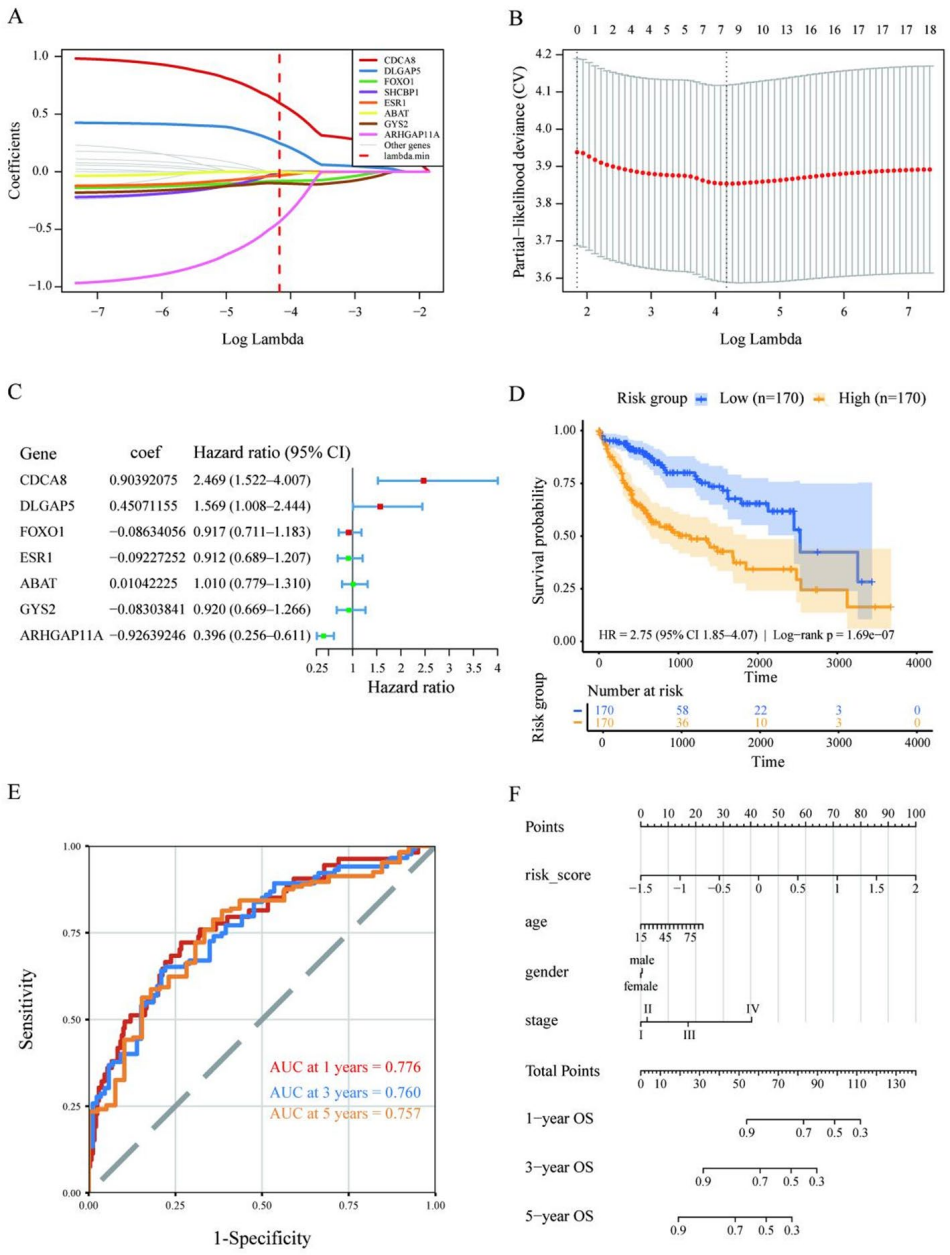
Construction and validation of the seven-gene prognostic model in the TCGA-LIHC cohort. **A** The LASSO–Cox regularization path plot shows the coefficient trajectories of the eight finally selected genes (colored) as a function of log(λ), with the vertical dashed line indicating the optimal λ value determined by cross-validation. **B** The LASSO–Cox cross-validation error curve shows the change in partial likelihood deviance with log(λ), with the vertical dashed line marking the selected optimal λ value. **C** The seven-gene prognostic signature identified by the LASSO–Cox model. The hazard ratios (HR) and 95% confidence intervals of CDCA8, ARHGAP11A, DLGAP5, GYS2, FOXO1, ESR1, and ABAT are shown. **D** Kaplan–Meier survival curves stratified by the median risk score (RS) calculated based on the seven-gene prognostic signature. The numbers of samples in each group, the hazard ratio (HR) with 95% confidence interval from the Cox proportional hazards model, and the *P* value from the log-rank test are shown. **E** Time-dependent ROC curves evaluating the predictive performance of the seven-gene signature for OS, with AUCs at 1, 3, and 5 years. The dashed line represents the line of no discrimination, and the AUC values at each time point are indicated in the lower-right corner. **F** A nomogram integrating the risk score (RS) with clinical covariates (age, sex, and stage). The point scale at the top is used to assign scores to each variable, and the total points correspond to individualized predictions of 1-, 3-, and 5-year OS, enabling clinically interpretable risk stratification and decision support

To evaluate the impact of feature genes on cancer prognosis, 20 hub genes were considered as candidate variables. Univariate Cox proportional hazards regression analysis was performed to assess the association between each gene and overall survival (OS), and 18 genes were selected for subsequent analysis (*P* value < = 0.05). Then, least absolute shrinkage and selection operator Cox (LASSO-Cox) regression was further applied to perform variable shrinkage and feature selection among the candidate genes. The regularization path of the LASSO-Cox model **(**Fig. 7A**)** showed that the regression coefficients of different genes gradually shrank with increasing values of the penalty parameter log(λ), with only a small subset of genes retaining non-zero coefficients over a relatively wide range of λ values. Subsequently, ten-fold cross-validation was used to evaluate the partial likelihood deviance under different λ values. The cross-validation error curve indicated that the model achieved the minimum deviance at the optimal λ (lambda.min), as shown in **(**Fig. 7B**)**, which was therefore selected as the final penalty parameter.

Under the optimal λ condition, the LASSO-Cox model ultimately selected seven genes to construct the prognostic risk model (*CDCA8*, *ARHGAP11A*, *DLGAP5*, *GYS2*, *FOXO1*, *ESR1*, and *ABAT*). Further Cox regression analysis was performed on this seven-gene signature, and the hazard ratios (HRs) of each gene were calculated, as shown in **(**Fig. 7C**)**. The results indicated that *CDCA8* and *DLGAP5* had positive regression coefficients (HR > 1) and acted as risk factors in the model, whereas *ARHGAP11A* had a negative regression coefficient (HR < 1) and exhibited a protective effect. The remaining four genes had HR values close to 1, indicating relatively modest risk effects.

Based on the median risk score, TCGA-LIHC patients were stratified into a high-risk group (*n* = 170) and a low-risk group (*n* = 170). Kaplan–Meier survival curves showed a significant difference in OS between the two groups (Fig. 7D) (HR = 2.75, 95% CI: 1.85–4.07; log-rank test *P* = 1.69 × 10⁻⁷). Time-dependent ROC curves were used to evaluate the predictive performance of the seven-gene signature at different follow-up time points **(**Fig. 7E**)**. The results showed that the AUCs for 1-year, 3-year, and 5-year OS were 0.776, 0.760, and 0.757, respectively, with a concordance index (C-index) of approximately 0.71, indicating that the model exhibited relatively stable discriminative ability in short- to mid-term follow-up. Furthermore, a nomogram was constructed by integrating the risk score with age, sex, and tumor stage **(**Fig. 7F**)**, which allowed the survival probabilities at 1, 3, and 5 years to be estimated by assigning scores to each covariate, thereby providing an intuitive tool for individualized prognostic assessment in HCC patients.

### Analysis of Molecular Subtypes for HCC

**Fig. 8:**
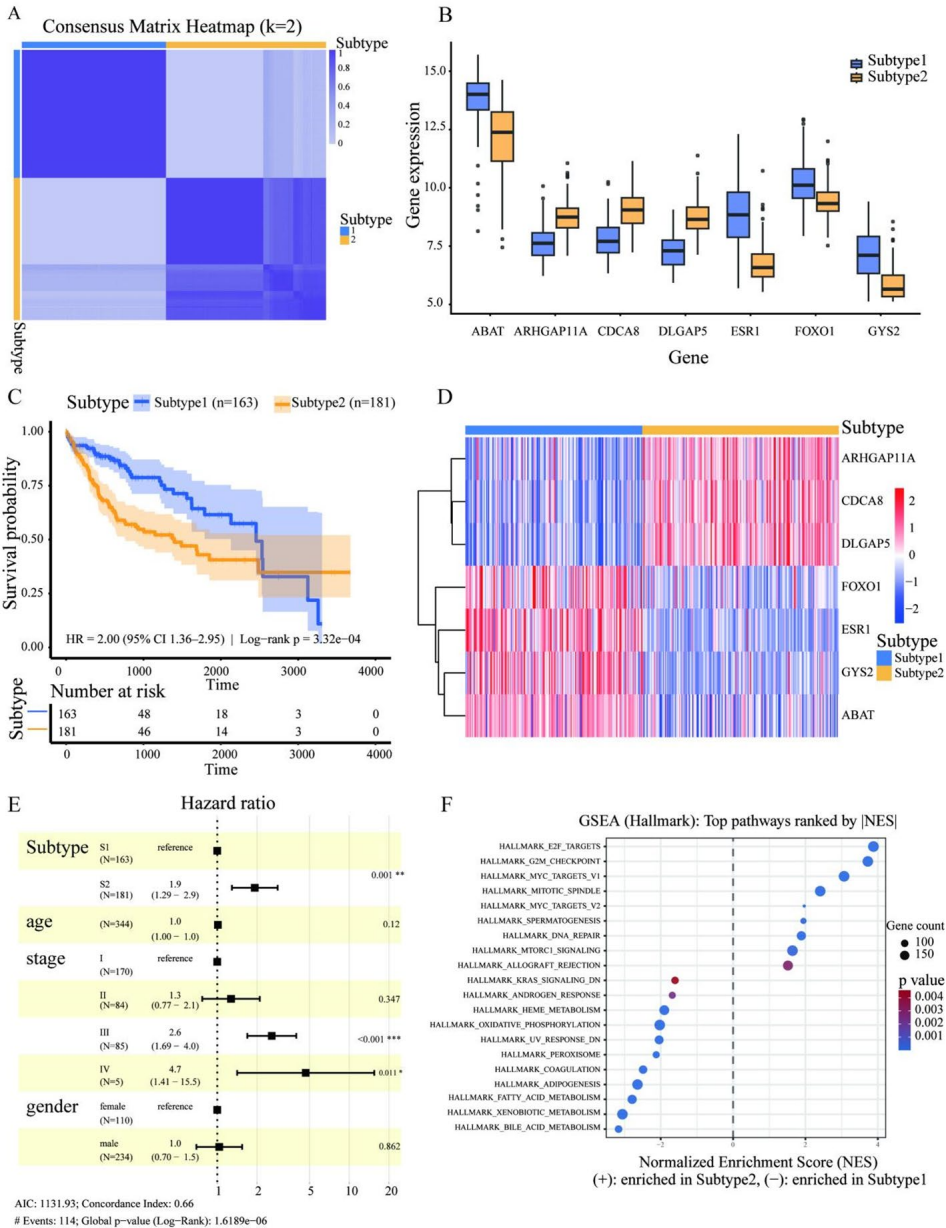
Seven-gene–based molecular subtypes of HCC and their prognostic and functional characteristics. **A** Consensus clustering consensus matrix heatmap (K = 2). The color bars on the top and right of the samples indicate the two subtypes. Heatmap colors range from light (low consensus) to dark (high consensus). **B** Box plots showing the expression of seven genes in Subtype 1 and Subtype 2, with blue representing Subtype 1 and yellow representing Subtype 2. **C** Kaplan–Meier survival curves for the two subtypes, with shaded areas indicating 95% confidence intervals and the number at risk shown below the curves. **D** Heatmap of the expression of seven genes in TCGA-LIHC samples, with columns representing samples and rows representing genes. The top annotation bar indicates subtypes. Colors range from blue to red, representing low to high relative expression levels. **E** Forest plot of a Cox proportional hazards regression model including subtype and clinical covariates (age, sex, and stage). Squares represent hazard ratios (HRs), horizontal lines indicate 95% confidence intervals, and the dashed vertical line denotes the null effect (HR = 1). **F** GSEA results for the two HCC molecular subtypes. The top 20 pathways ranked by the absolute value of the normalized enrichment score (NES) are shown. NES > 0 indicates pathways enriched in Subtype 2, whereas NES < 0 indicates pathways enriched in Subtype 1. Bubble size represents the number of genes, and color indicates the nominal *p* value

Based on the seven genes, we performed unsupervised molecular subtyping of the TCGA-LIHC cohort using a consensus clustering approach and determined the optimal number of clusters as *k* = 2. The consensus matrix heatmap showed high intra-subtype consistency and clear separation between the two subtypes (Fig. 8A), indicating good stability of the identified classification. Accordingly, samples were divided into Subtype 1 (*n* = 163) and Subtype 2 (*n* = 181). As shown in (Fig. 8B, D), the expression levels of the seven genes differed markedly between the two molecular subtypes: *CDCA8*, *ARHGAP11A*, and *DLGAP5* were generally more highly expressed in Subtype 2, whereas *GYS2*, *FOXO1*, *ESR1*, and *ABAT* showed relatively higher expression in Subtype 1.

Kaplan–Meier survival analysis revealed a significant difference in OS between the two subtypes (Fig. 8C), with Subtype 2 exhibiting a markedly poorer prognosis than Subtype 1 (hazard ratio (HR) = 2.00, 95% confidence interval (CI): 1.36–2.95; log-rank *P* = 3.32 × 10⁻⁴). To evaluate whether this molecular classification served as an independent prognostic factor, the subtype variable was further incorporated into a multivariate Cox proportional hazards model together with age, sex, and tumor stage (Fig. 8E). The results showed that Subtype 2 remained significantly associated with an increased risk of death (HR = 1.90, 95% CI: 1.22–2.90, *P* ≤ 0.001). In addition, advanced tumor stage (stages III and IV) exhibited a higher mortality risk compared with early-stage disease (stage I). These findings indicated that the molecular subtypes possess independent prognostic predictive value beyond traditional clinical features such as tumor stage.

To elucidate the function of molecular subtypes, gene set enrichment analysis (GSEA) based on the Hallmark gene sets was performed(Fig. 8F). The results showed that the poorer-prognosis Subtype 2 (NES > 0) was significantly enriched in pathways related to cell cycle progression and proliferation, including E2F targets, G2M checkpoint, mitotic spindle, MYC targets, as well as DNA repair and mTORC1 signaling. These pathways are closely associated with enhanced tumor cell proliferation and growth signaling and have been repeatedly reported in previous HCC studies to correlate with aggressive phenotypes and unfavorable outcomes [59, 60]. In contrast, the better-prognosis Subtype 1 (NES < 0) was mainly enriched in metabolism- and hepatocyte function–related pathways, such as bile acid metabolism, xenobiotic metabolism, peroxisome, and oxidative phosphorylation, suggesting that this subtype retained features of metabolic homeostasis [61].

### Immune Infiltration Characteristics and Immune Checkpoint Analysis

**Fig. 9:**
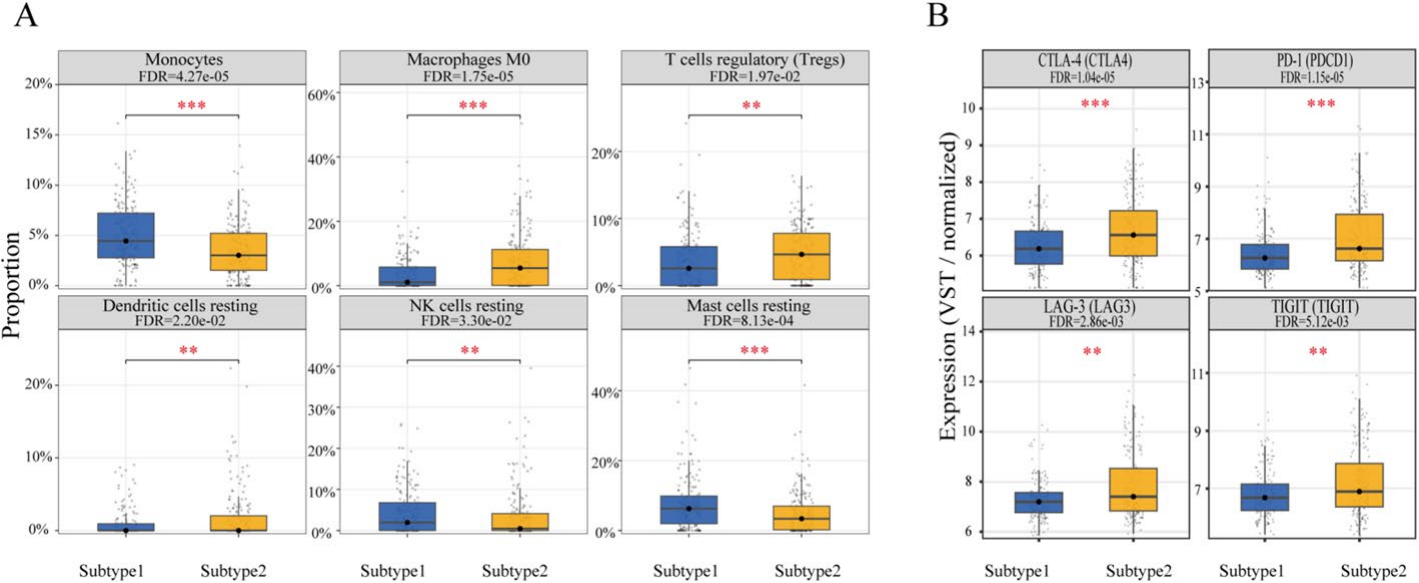
Immune infiltration patterns and immune checkpoint expression in the two molecular subtypes. **A** Comparison of immune cell composition between the two subtypes. Blue represents Subtype 1, and yellow represents Subtype 2. **B** Comparison of the expression of four immune checkpoint genes (PDCD1, CTLA4, LAG3, and TIGIT) between the two subtypes

Based on immune cell infiltration profiles estimated by CIBERSORT, differences in the immune microenvironment between the two molecular subtypes were compared (Fig. 9A). The results showed that multiple immune cell types differed significantly between Subtype1 and Subtype2 (FDR ≤ 0.05). Specifically, Subtype1 exhibited relatively higher infiltration proportions of monocytes, resting dendritic cells, and resting mast cells, whereas Subtype2 was characterized by increased proportions of M0 macrophages, regulatory T cells (Tregs), and resting natural killer (NK) cells. These differences indicated that the two subtypes differed markedly in immune cell composition as well as in inflammatory and immunosuppressive states [62].

We further compared the expression levels of representative immune checkpoint molecules, including *CTLA4*, *PD-1*, *LAG3*, and *TIGIT* (Fig. 9B). The results showed that all four genes were significantly upregulated in Subtype2 (all FDR ≤ 0.01). In addition to the enrichment of immunosuppression-associated immune cell components (such as M0 macrophages and Tregs), Subtype2 also exhibited higher expression of immune checkpoint molecules, suggesting that it may reside in a more pronounced immunosuppressive or “exhausted” immune state [63, 64].

### Validation of Prognosis in Independent Datasets

**Fig. 10:**
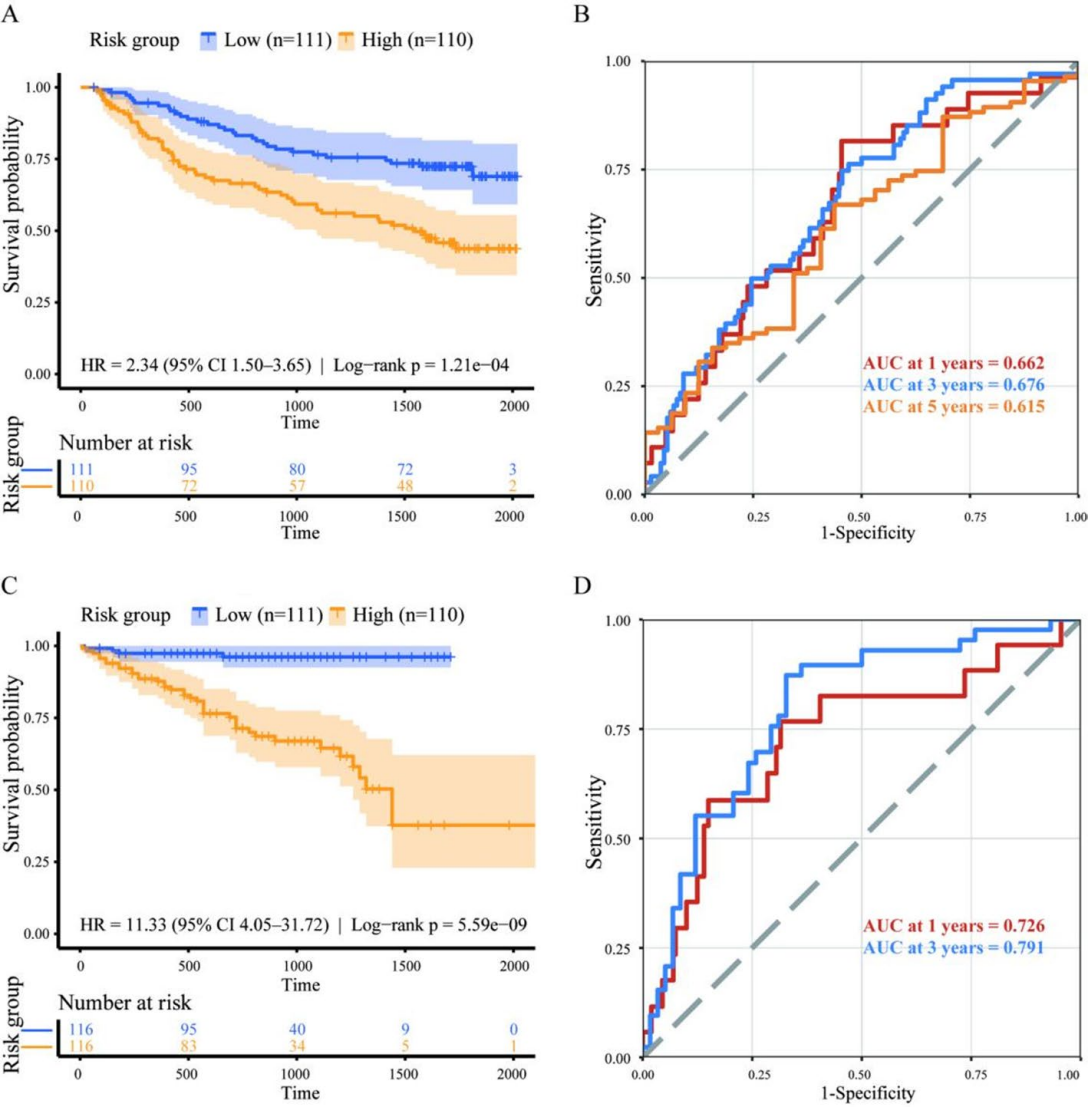
Validation of the prognostic performance of the seven-gene signature in independent cohorts. **A** Kaplan–Meier curves of the GSE14520 cohort (blue indicates low risk, yellow indicates high risk). **B** Time-dependent ROC curves of the GSE14520 cohort. **C** Kaplan–Meier curves of the ICGC-LIRI-JP cohort (blue indicates low risk, yellow indicates high risk). **D** Time-dependent ROC curves of the ICGC-LIRI-JP cohort

After applying the seven-gene prognostic signature to the GSE14520 cohort, patients were stratified into high- and low-risk groups according to the median risk score. Kaplan–Meier survival analysis showed a significant difference in OS between the two groups (Fig. 10A)(hazard ratio [HR] = 2.34, 95% confidence interval [CI]: 1.50–3.65; log-rank *P* = 1.21 × 10⁻⁴). ROC analysis further demonstrated that the signature achieved AUC values of 0.662, 0.676, and 0.615 for predicting 1-, 3-, and 5-year OS, respectively (Fig. 10B), indicating that the prognostic signature retained stable discriminative ability in an independent population.

In the ICGC-LIRI-JP cohort, patients were also stratified into high- and low-risk groups according to the median value. The survival curves of the two groups were more distinctly separated (Fig. 10C)(HR = 11.33, 95% CI: 4.05–31.72; log-rank *P* = 5.59 × 10⁻⁹). Time-dependent ROC analysis showed that the model achieved AUC values of 0.726 and 0.791 for 1-year and 3-year survival prediction, respectively (Fig. 10D), indicating good predictive performance during early follow-up. It should be noted that in the ICGC-LIRI-JP cohort, only 4 of 116 patients (3.4%) in the low-risk group experienced death events during follow-up, resulting in a high censoring rate of 96.6%. Consequently, the number of events at later follow-up time points was extremely limited, leading to substantial uncertainty in the estimation of the 5-year AUC; therefore, this metric was not interpreted as a primary evaluation index. Overall, across the two independent cohorts, GSE14520 and ICGC-LIRI-JP, the seven-gene signature consistently and effectively discriminated between high- and low-risk populations, supporting its reproducible prognostic predictive value.

## Conclusion

This study focused on miRNA–disease association prediction and the identification of HCC-related molecular biomarkers, and proposed and systematically evaluated the adaptive multi-order moment model MAGMDA. MAGMDA exhibited stable and competitive predictive performance in terms of metrics such as AUC and AUPR, while reducing reliance on fixed moment orders and manually defined thresholds. In addition, MAGMDA introduced interpretable diagnostic signals for moment-order selection, which improved the transparency of high-order statistical modeling.

Compared with traditional high-order statistical models that relied on fixed moment orders and empirically defined thresholds, MAGMDA adopted a learnable gating mechanism to replace static configurations. This mechanism integrated Gumbel–Softmax sampling, a dynamic threshold network, and a Top-K retention strategy to automatically select a limited set of moment orders within a predefined maximum order. Subsequently, a cross-order attention layer assigned differentiated weights to the selected orders, moving beyond uniform averaging. Mechanistic analysis, including the selection frequency of moment orders and the distribution of attention weights, revealed a consistent preference for a limited subset of key orders across folds and layers. These observations suggest that the adaptive modules function as effective structural filters rather than redundant components.

At the biological level, this study used HCC as an application scenario and started from the HCC-related miRNAs predicted by MAGMDA. By integrating external database validation, differential expression analysis, miRNA–mRNA negative regulatory network construction, functional enrichment analysis, and PPI hub gene identification, we identified candidate genes associated with cell cycle regulation, metabolic reprogramming, and extracellular matrix remodeling. These findings are consistent with established characteristics of HCC. Based on this gene set, a seven-gene prognostic signature was constructed, which exhibited stable survival stratification ability in the TCGA-LIHC cohort and was successfully validated in two independent cohorts, GSE14520 and ICGC-LIRI-JP. These results indicated that the molecular networks inferred by MAGMDA were statistically robust and generalizable across independent populations. Molecular subtyping and immune infiltration analysis revealed systematic differences across subtypes in proliferation, metabolism, transcription, immunosuppressive cell components, and immune checkpoint expression, providing additional insights into HCC heterogeneity and its immune microenvironment.

Under fair and comparable evaluation settings, the proposed MAGMDA achieved stable predictive performance together with favorable interpretability. Through multi-level bioinformatics validation using HCC as an example, this study further demonstrates the potential of MAGMDA in miRNA–disease association discovery and tumor prognosis research.

### Limitations and Future Directions

Although MAGMDA demonstrated stable performance under the five-fold cross-validation framework, several limitations should be acknowledged. In this study, GIP similarity matrices were constructed using the complete set of experimentally validated associations in HMDD v2.0 prior to cross-validation partitioning. While this strategy follows common practice in miRNA–disease association prediction studies and treats similarity matrices as prior structural knowledge, the similarity structure implicitly reflects all known associations. Therefore, a mild optimistic bias cannot be completely excluded. In future work, recomputing association-dependent similarity matrices within each training fold may further eliminate potential information leakage and provide a more strictly inductive evaluation setting.

In addition, MAGMDA is primarily designed under a transductive learning framework, where diseases and miRNAs in the prediction task are assumed to be partially observed in the known association network. In strict cold-start scenarios, where a disease has no known miRNA associations in the training set, the interaction-based similarity component (e.g., GIP) becomes less informative. Although ontology-based disease semantic similarity can still provide biologically meaningful prior information, predictive performance may decrease in such settings. Extending the framework to better support completely unseen diseases remains an important direction for future research.

Furthermore, the negative samples used in this study were randomly drawn from the set of unconfirmed miRNA–disease pairs due to the lack of a reliable gold-standard negative dataset. Although this strategy is widely adopted in current MDA prediction studies, some unconfirmed pairs may correspond to yet-undiscovered true associations. Consequently, sampling-induced bias cannot be entirely ruled out. Future work could explore more conservative negative sampling strategies or semi-supervised learning frameworks to mitigate this limitation.

Although a multi-level validation framework combining database references and independent transcriptomic cohorts was employed, some degree of confirmation bias inherent to database-supported biomedical research cannot be entirely excluded. Future studies incorporating prospective experimental validation would further strengthen the biological interpretability and translational value of the findings.

## Data Availability

The known miRNA–disease association data used in this study were obtained from the Human microRNA Disease Database (HMDD, version 2.0). Transcriptomic and corresponding clinical data for liver hepatocellular carcinoma (TCGA-LIHC) were downloaded from The Cancer Genome Atlas (TCGA) program. Two independent external validation cohorts, GSE14520 and ICGC-LIRI-JP, were retrieved from the Gene Expression Omnibus (GEO) and the International Cancer Genome Consortium (ICGC), respectively. All datasets analyzed in this study are publicly available from their respective repositories.The source code and related resources for the proposed MAGMDA framework are publicly available at https://github.com/zhangclbio/MAGMDA.

## Abbreviations

MAGMDA: A multi-order adaptive graph-based miRNA-disease association model
MDA: miRNA-disease associations
GNN: Graph neural network
PPI: Protein–protein interaction
HCC: Hepatocellular carcinoma
TCGA: The Cancer Genome Atlas
GEO: Gene Expression Omnibus
ICGC: International Cancer Genome Consortium
